# A guide to functionalisation and bioconjugation strategies to surface-initiated polymer brushes

**DOI:** 10.1039/d3cc01082a

**Published:** 2023-05-09

**Authors:** Carlos Eduardo Neri-Cruz, Franciane Mouradian Emidio Teixeira, Julien E. Gautrot

**Affiliations:** a School of Engineering and Materials Science, Queen Mary University of London Mile End Road London E1 4NS UK j.gautrot@qmul.ac.uk; b Department of Immunology, Institute of Biomedical Sciences, University of São Paulo São Paulo 05508000 Brazil

## Abstract

Since the first introduction of their concept in the 1980s and 90s, polymer brushes have been the focus of intense research efforts to identify novel physico-chemical properties and responsiveness, and optimise the properties of associated interfaces for an ever growing range of applications. To a large extent, this effort has been enabled by progress in surface initiated controlled polymerisation techniques, allowing a huge diversity of monomers and macromolecular architectures to be harnessed and achieved. However, polymer functionalisation through chemical coupling of various moieties and molecular structures has also played an important role in expanding the molecular design toolbox of the field of polymer brush science. This perspective article reviews recent progress in polymer brush functionalisation, discussing a broad range of strategies for the side chain and end chain chemical modification of these polymer coatings. The impact of the brush architecture on associated coupling is also examined. In turn, the role that such functionalisation approaches play in the patterning and structuring of brushes, as well as their conjugation with biomacromolecules for the design of biofunctional interfaces is then reviewed and discussed.

## Abstract 2

This review presents recent advances in surface initiated polymer brush functionalisation, as a guide for the design of functional interfaces.

## Graphical abstract

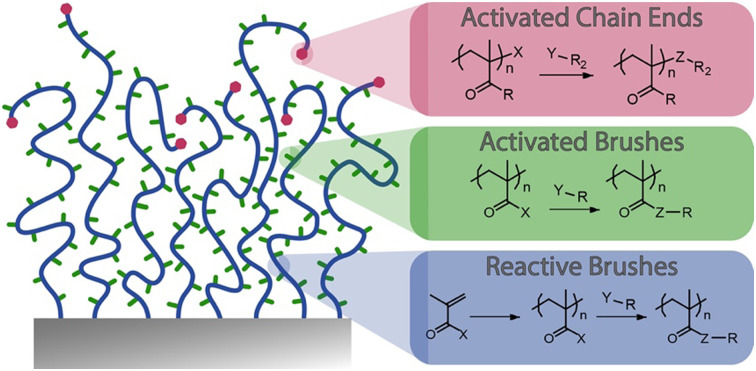

## Introduction

Polymer coatings have been broadly applied to control the surface properties of most types of substrates and materials, and enhance their performance for application and translation. In particular, when these coatings are generated *via* a surface initiated controlled polymerisation mechanism, dense polymer monolayers can be generated that display unique physico-chemical properties. These interfaces, in which polymer chains are grafted from underlying substrates at high densities, are known as polymer brushes.^1–4^ Development and optimisation of controlled radical polymerisation and ring opening polymerisation techniques have enabled the production of a very wide range of polymer brushes with varying chemistries and controlled physico-chemical properties. In addition, the flexibility with which the grafting density of resulting polymer brushes can be controlled, over a wide range (typically from below 0.1 to 0.7 chains nm^−2^), enables the control of a transition in morphology from isolated chains (“mushroom” regime) to densely crowded and stretched chains (“brush” regime).^5^

These systems find applications in very varied fields, from catalysis and electronic devices to biosensing and tissue engineering.^6–9^ Key to translation is the ability to control and optimise functional properties of polymer brushes and associated materials and interfaces. For example, the high surface density of polymer brushes and responsive behaviour of some of these coatings has been applied to the design of novel catalytic systems, or to control the stability of colloids.^10–12^ In the biomedical field, the ability to generate dense, yet thin hydrophilic polymer coatings with well-defined physico-chemical properties (*e.g.* surface charge, thickness, balance of hydrophilicity) has enabled their application to design biosensors, or for the engineering of scaffolds and nanomaterials for tissue engineering and regenerative medicine.^9^ This includes mediating cell or tissue bonding, or to design implant coatings, cell based assays and gene delivery systems.^13–20^

**Fig. 1:**
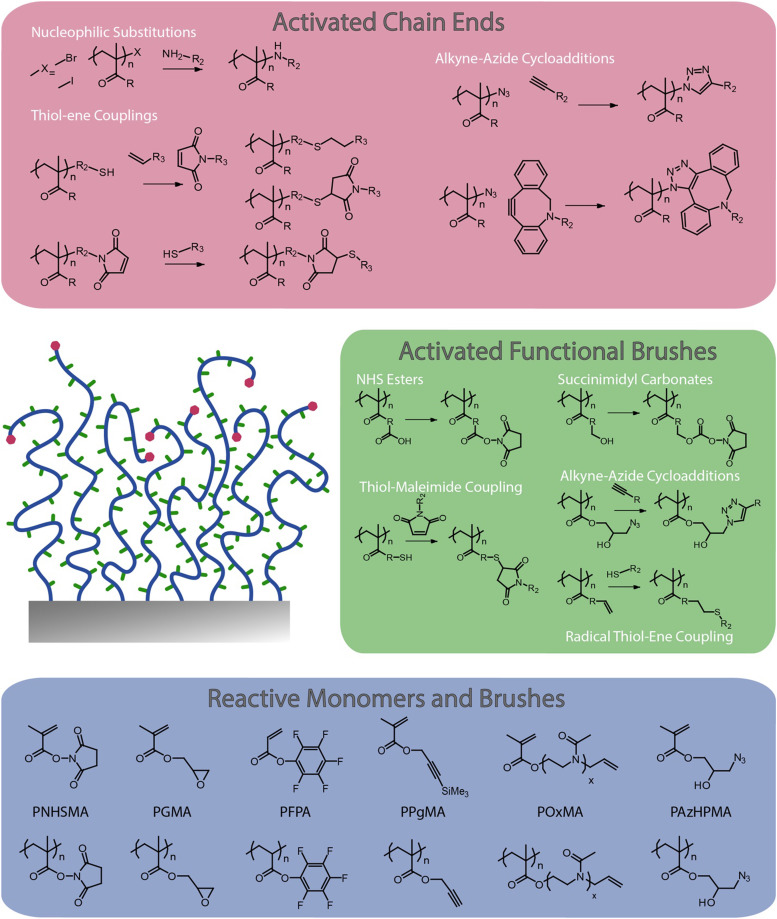
Selected examples of polymer brush functionalisation strategies: side chain and end chain functionalisation, and examples of chemically reactive polymer brushes and corresponding monomers.

Although some inherently functional polymer brushes have been reported, for example to promote electron transfer or to confer antibacterial properties,^21,22^ in most cases the functional properties and performance of brushes are achieved through the coupling of chemical moieties to usual polymer brushes. Unlike polymer coatings generated *via* a “grating to” approach, which allows functionalisation of precisely designed and characterised macromolecular structures^23–25^ prior to coupling to a surface (but at low density), polymer brushes generated *via* a grafting from approach, often using surface-initiated radical polymerisation techniques,^3,4,26^ are more challenging to functionalise, owing to the steric hindrance associated with dense chain packings. To do so, a broad range of chemical strategies have been applied to the functionalisation of polymer brushes. In this respect, these coatings offer unique opportunities for the precise engineering of interfaces with well-defined chemistry and hierarchical nanostructure. Indeed, polymer brush functionalisation can be mediated through their side chains, as well as specifically to end chains (Fig. 1). In addition, the ability to pattern polymer brushes, at different length scales, and the simplicity with which their architecture can be structured in the *z*-direction, for example through block copolymer brush formation, offers unique opportunities to the precise design of physico-chemical properties and optimisation of functional performance.

This perspective article reviews the broad range of coupling strategies that have been proposed to confer functionality to polymer brushes (Fig. 1). It focuses specifically on the chemical functionalisation of polymer brushes generated *via* a grafting from approach and surface-initiated controlled radical polymerisation techniques, as this strategy enables accessing both dense and sparse polymer brush coatings. It should be noted however that concepts and strategies discussed for low density brushes can be applied to a broad range of other polymer coatings, for example generated *via* a “grafting to” approach, or for hydrogel functionalisation. The various chemistries that have been explored to functionalise the side chains of polymer brushes are first discussed, and the impact of the brush density and architecture on such processes is examined. Approaches to selectively functionalise end chains of polymer brushes are then presented. The application of these tools to the chemical patterning of brushes are then reviewed, briefly presenting recent progress in brush patterning, but mainly focusing on the functionalisation of patterned brushes at the nano-to-microscale. The structuring of the brush chemistry in the *z*-direction (*z*-structuring), perpendicular to the plane of the substrate, is also discussed. Finally, the application of functionalisation strategies for the conjugation of peptides and proteins to brushes is examined, presenting guidelines to the rationale design and selection of coupling strategies towards biofunctional polymer brushes.

## Side chain functionalisation strategies

Side-chain functionalization is commonly used to produce brushes with high functionalisation degrees (Table 1). This may result in coatings with increased chemical affinity or functionality.^27^ In turn, the associated surface density in functional groups can be readily controlled through the design of the brush architecture (*e.g.* thickness and degree of polymerisation, copolymerisation with other functional/non-functional monomers and grafting density)^28–30^ Post-polymerisation functionalisation is particularly attractive owing to the difficulty of synthesising and controlling the surface initiated polymerisation of functional monomers, in particular with moderate to large molar masses. However, ensuring efficient coupling to side chains, in particular throughout the entire backbone, including inner brush layers, is commonly challenging,^31,32^ owing to the inherent crowding and steric hindrance associated with deeper functionalisation of side chains.^33,34^ In addition, direct side-chain functionalization may potentially lead to side reactions, such as the cross-linking of polymer brushes^31^ and their branching,^35^ potentially impacting their physico-chemical properties. Therefore effective strategies to side-chain functionalisation of polymer brushes are essential to the optimisation of the properties and performance of these coatings for a range of applications.

**Table 1:** Selected examples of functionalisation strategies to conjugate molecules to the side chains of polymer brushes

Brush type	CRP	Architecture	Post-polymerization modification strategy	Molecule(s) coupled	DF	Application	Ref.
PCBMAA-PHPMAA	ATRP	Random copolymer	Amide bond formation using NHS-ester	RGD peptide	n.s.	Cell culture	36
		*h* = n.s.					
PDEGMA–PSCEMA	RAFT	Random copolymer	Amide bond formation using DSC/NHS	4-(Trifluoromethyl)benzyl amine and biotin	80%	Biosensing	31
PDEGMA–PNHSMA		*h* = 41 ± 1 nm			6%		
POEGMA-*b*-PHEMA	ATRP	*h* = 1–20 nm	Amide bond formation using NHS-ester	IgG and bovine serum albumin (BSA)	n.s.	Biosensing	28
PMAA	ATRP	n.s.	Amide bond formation using NHS-ester	3-Aminophenylboronic acid	n.s.	Biosensing	48
PAA	ATRP	*h* = 200 nm (by SEM)	Amide bond formation using NHS-ester	l-Leucine methyl ester	70%	na	27
PMAA	ATRP	n.s.	Amide bond formation using NHS-ester	3-Amino-1-azide propane	62%	n.s.	34
PAA	ATRP	*h* = 55 nm	Amide bond formation using NHS-ester	*N*,*N*-Bis(carboxymethyl)-l-lysine (NTA)	n.s.	Biosensing	49
PMAA	ATRP	*h* = 180 nm	Amide bond formation using NHS-ester	*N*,*N*-Bis(carboxymethyl)-l-lysine (NTA)	n.s.	Biosensing	50
PHPMAA-PCBMAA-PSBMAA	ATRP	*h* = 50 ± 10 nm	Amide bond formation using NHS-ester	Immunoglobulin G (IgG) antibodies	n.s.	Biosensing	56
PHEMA	ATRP	h = 56 nm	Carboxylation and amide bond formation using NHS-ester	Gelatin	n.s.	Cell culture	37
		*σ* = 0.6 chain nm^−2^					
PCEA	Not CRP	Homopolymer	Amide bond formation using NHS-ester	*N*,*N*-Bis(carboxymethyl)-l-lysine (NTA)	n.s.	Antimicrobial coatings	38
PCEA-*b*-PHEMA	Not CRP	Block copolymer	Amide bond formation using NHS-ester formation	Dimethylmaleic anhydride/antimicrobial peptide			39
PEDA-*b*-PHEMA		*h* ∼ 20 μm (CLSM)		Bovine serum albumin	n.s.	Antimicrobial coatings	
POEGMA	ATRP	*h* = 107 ± 13 nm	Carboxylation and amide bond formation using NHS-ester	RGD (GRGDSPC) and RGD-FITC	n.s.	Cell culture	40
PHPMA-*co*-CBMAA	SET-LRP	*h* = 30–40 nm	Amide bond formation using NHS-ester	Tissue plasminogen activator (tPA)	n.s.	Antifouling surfaces	57
POEGMA	ATRP	n.s.	Amide bond formation using NHS-ester	BSA serum albumin (BSA)	n.s.	n.s.	41
PDMA-PAPMA	ATRP	Random copolymer	Thiol-maleimide coupling	Cysteine-terminated HHC-36 (KRWWKWWRR) antimicrobial peptide	n.s.	Antimicrobial coatings	42
PDMAPS-*b*-PMAA	ATRP	Block copolymer	Amide bond formation using triazine-ester	HHC-36 antimicrobial peptide	n.s.	Antimicrobial coating	43
		*h* = 170 ± 5 nm					
Poly(γ-*tert*-butyl-l-glutamic acid)-*b*-poly(sarcosine)	Not CRP	Block copolymer	Amide bond formation using triazine-ester	Dopamine	56–65%	Antimicrobial coating	58
	ROP	*h* = 1.8–4.5 nm					
		*σ* = 0.03–0.13 chain nm^−2^					
PFPA	RAFT	Homopolymer	Amide bond formation *via* nucleophilic addition to pentafluorophenyl ester	Anti-PKR antibody	n.s.	Bio-separation	32
		*σ* = 0.11 chain nm^−2^					
PFPA	RAFT	Homopolymer	Amide bond formation *via* nucleophilic addition to pentafluorophenyl ester	Spiropyran-amine	n.s.	Patterning	59
		*h* = 12–16 nm		5-((2-Aminoethyl)amino)naphthalene-1-sulfonic acid			
		*σ* = 0.3 chain nm^−2^					
PTFAMA	Not CRP	Homopolymer	Amide bond formation *via* nucleophilic addition to pentafluorophenyl-activated esters	Spiropyran-amine	40–50%	Separation	60 and 61
PGMA		*h* > 100 nm	Nucleophilic substitution to epoxides		90–95%		
PGMA-*co*-PDEAEMA	ATRP	*h* = 190–240 nm	Nucleophilic substitution to epoxides	Bovine serum albumin and lysozyme	48–79%	Biosensing	29
PDMAEMA-*co*-P(AzHPMA)	ATRP	*h* = 150–250 nm	Copper(i)-catalyzed azide–alkyne cycloaddition	*S*-Propargyl thioacetate	100%	Reversibly cross-linked brushes	35
PGMA	ATRP	74 ± 3 nm	Nucleophilic substitution to epoxides	Thiols	100%	Reversibly cross-linked brushes	62
PGMA	ATRP	*h* = 53 ± 7 nm	Nucleophilic substitution to epoxides and copper(i)-catalysed azide–alkyne cycloaddition	Propargylic ferrocene carboxylate	100%	Responsive polymer brushes	63
		*σ* = 2.6 ± 0.4 chain nm^−2^					
POEGMA	ATRP	*h* ≈ 20 nm	Ether formation using boron trifluoride-ether complex	β-d-Maltose octoacetate	n.s.	Cell culture	64
		*σ* ≈ 0.7 chain nm^−2^					
PHEMA	ATRP	*h* ∼ 9–26 nm	Carbodiimide-mediated esterification	*o*-Nitrobenzyl thioether	77 ± 2%	n.s.	30
			Thiol–ene coupling	*p*-Methoxyphenacyl thioether	88 ± 3%		
			Isocyanate coupling	Dodecyl isocyanate, furfuryl isocyanate, adamantyl isocyanate, and cyanophenyl maleimide.			
PNIPAM-PAA	ATRP	*h* ∼ 21 nm	Amide bond formation using NHS-ester	RGD peptide (GRGDS)	n.s.	Cell adhesion	65
PPgMA	Not CRP	n.s.	Thiol–yne coupling	Range of thiols	n.s.	Photopatterning of brush chemistry	66
PGMA	ATRP	*h* ∼ 20 nm	Thiol–ene coupling	Range of thiols	1–76%	Protein patterning	67
POEGMA	ATRP	*h* ∼ 60 nm	Thiol–ene coupling	RGD peptide	n.s.	Cell patterning	68
POEGMA	ATRP	*h* ∼ 40 nm	Thiol–ene coupling	Cell adhesive peptides	6–51%	Cell adhesion and patterning	69
				Acetyl cysteine			
				Glutathione			
POEGMA	ATRP	*h* = 10–100 nm	Schiff base chemistry	Histidine	n.s.	Protein and tissue adhesion	70
PHEMA	ATRP	*h* ∼ 145 nm	Diazirin photoactivation	Coupling to tissue (bovine meniscus)	n.s.	Tissue bonding	14
POEGMA	ATRP	*h* ∼ 36 nm	Tetrazole coupling (acyl chloride), followed by phototriggered nitrile imine-mediated tetrazole-ene cyclocloaddition	Maleimides (biotin or ATRP initiator)	n.s.	Bioconjugation and photopatterning	71

### Reactive esters for amide bond formation

Amide bond formation, starting from carboxylated or primary amine-functional brushes remains the most common side chain functionalisation strategy investigated. Owing to the low reactivity of carboxylic acids for amidation, brushes displaying reactive esters are typically generated to achieve high densities of amine coupling. Carboxyl groups present in polymer brushes based on poly(acrylic acid) (PAA) and poly(methacrylic acid) (PMAA), can readily produce reactive esters upon reaction with corresponding activating agents such as *N*-hydroxysuccinimide/1-ethyl-3(3-dimethylaminopropyl) carbodiimide (NHS/EDC)^36–42^ and triazines,^43^ subsequently allowing further coupling of amines (in particular primary amine-containing molecules).^34^

Carboxylic acid activation has predominantly relied on the use of coupling agents based on carbodiimide and succinimide chemistries, alone or in combination, since this provides relatively high conversion efficiencies.^27,34^ When EDC was used alone, reaction with carboxylic acids produced *O*-acylisourea intermediates, which can then quickly react with primary amines.^27^ However, conversion rates remain typically low, since carboxyl groups can also be regenerated *via* direct hydrolysis of *O*-acylisourea intermediates.^27,44^ Activation of carboxylic acids into esters and specificity towards amide formation rather than hydrolysis is therefore a key aspect that has to be controlled in order to achieve high degrees of functionalisation.

**Fig. 2:**
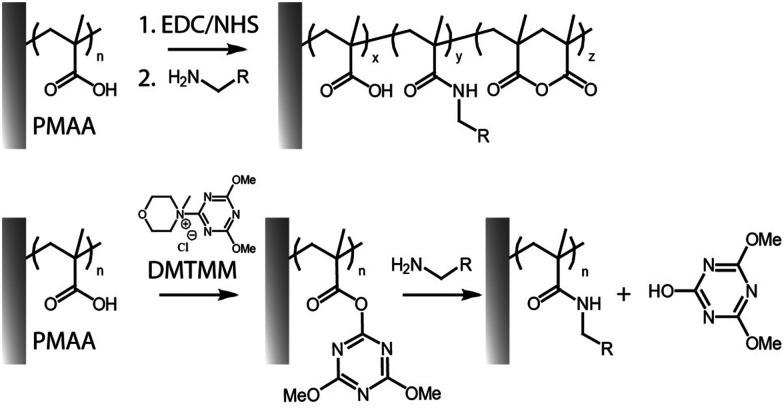
Activation of PMAA brushes with EDC/NHS can result in a mixture of amide, anhydride and carboxylic functions on the brush backbone (top). Coupling of amines to PMAA brushes using DMTMM as activating reagent (bottom).

More controlled and highly efficient coupling reactions involve the use of NHS and its derivatives. NHS is commonly used to stabilize the *O*-acylisourea esters, leading to the formation of more stable intermediates. The resulting compounds are modestly stable in dry conditions, and have a half-life in aqueous buffers, at near neutral pH and room temperature, of 10 min (reported for poly(carboxybetaine) brushes).^45,46^ While EDC/NHS coupling is the standard route for functionalisation, it is typically associated with modest efficiencies, in particular when relatively high concentrations of amines are not practical,^32^ as well as due to its dependence on pH.^47^ Indeed, as protonated amines cannot enable amidation of NHS-activated esters, weakly alkaline pH are required to promote coupling, but may result in some hydrolysis.^34,47^ In addition, unlike coupling reactions involving monomeric acids and amines, EDC/NHS activated carboxylate brushes can rearrange rapidly to form anhydrides (Fig. 2).^34^ Although such species remain potentially active for amidation, functionalisation levels can be further enhanced through series of activation–amidation cycles, ultimately achieving functionalisation levels as high as 62%, in the case of 3-amino-1-azidopropane, and 70% with l-leucine methyl ester.^27,34^ The respective levels of anhydride, *N*-acylurea and amide formation (quantified through Fourier transform infrared spectroscopy) was found to be affected by the type of brush investigated, and the concentration and ratio of reagents used.

As a result of its reasonable efficiency and the simplicity of this functionalisation strategy, EDC/NHS coupling has been used widely for the functionalisation of poly(acrylic acid) (PAA) and poly(methacrylic acid) (PMAA) brushes. For example, 3-aminophenylboronic acid was coupled to PMAA brushes *via* EDC/NHS coupling, to enable the capture and detection of glucose (dynamic sensing range at mM concentrations).^48^ Similarly, *N*,*N*-bis(carboxymethyl)-l-lysine (NTA), a ligand used to chelate Ni^2+^ ions and capture histidine-tagged (His-tagged) proteins, was tethered to PAA and PMAA brushes *via* EDC/NHS coupling.^49,50^ Coupling of NTA residues was also achieved through the functionalisation of poly(carboxyethyl acrylate) brushes using the same approach.^38^ After coordination of cerium(iv) ions, this allowed mimicking of DNAse catalytic activity to limit bacterial adhesion and biofilm formation.

In a number of cases, polymer brushes lacking carboxylate terminated side chains can be functionalised prior to further coupling with EDC/NHS. This is the case of poly(hydroxyethyl methacrylate) (PHEMA), (poly(oligoethylene glycol methacrylate) (POEGMA),^40^ and POEGMA-*co*-PHEMA, using succinic anhydride and a base (*e.g.* dimethylaminopyridine) as catalyst^28,40,51–53^ Residual active NHS-esters can disrupt the antifouling properties of activated polymer brushes, thus exposure to deactivators, such as amino compound bearing carboxyl or sulfate groups can be carried out.^54^ An alternative strategy consists in functionalising amine-bearing brushes with reactive esters. For example, maleimide-NHS esters have been used to functionalize PDMA-*co*-APMA brushes with maleimide moieties prior to further coupling.^42,55^ Indeed, the heterobifunctional sulfosuccinimidyl 4-(*N*-maleimidomethyl)cyclohexane-1-carbonate) (Sulfo-SMCC) crosslinker containing a sulfo-NHS ester group at one end and a maleimide group has been widely used too, for example for the coupling of the cysteine-terminated HHC-36 (KRWWKWWRR) antimicrobial peptide.^42,55^ However, the use of amine side chains introduces high charge densities that limit protein resistance.

Alternatives to carbodiimides include triazines such as 4-(4,6-dimethoxy-1,3,5-triazin-2-yl) (DMTMM, Fig. 2), which has been applied to the modification of the carboxyl groups in polysaccharides, polypeptides, polyacrylic acid and polymethacrylic acid, conferring high yields and control over functionalization.^72,73^ DMTMM and corresponding reactive ester intermediates are soluble and more stable against hydrolysis, compared to those based on EDC, therefore enabling more effective amide bond formation in a variety of protic solvents.^47,74^ Though DMTMM has been used for amidation in a wide range of systems (*e.g.* to enable “grafting to” of free polymer chains onto surfaces, molecular polymer brushes and free polymer chains),^75–78^ only a few recent reports have explored this approach for the functionalization of surface-tethered polymer brushes.^43,58^ For example, the HHC-36 antimicrobial peptide was coupled to the carboxyl side chains of poly(3-[dimethyl-[2-(2-methylprop-2-enoyloxy)ethyl]azaniumyl]propane-1-sulfonate)-poly(methacrylic acid) (PDMAPS-*b*-PMAA) brushes, generated *via* SI-ATRP from the surface of polyurethane catheters, to prevent biofilm and thrombus formation.^14,79^ For similar applications, poly(γ-*tert*-butyl-l-glutamic acid)-*b*-poly(sarcosine) brushes were grafted from titanium dioxide surfaces. Conjugation with dopamine *via* amide bond formation, with 56–65% conversion, enabled the formation of silver particles at corresponding surfaces, upon reduction of silver ions by catechol functions.^58^ The use of such hierarchical structures displaying one component providing antifouling properties, while the other provides sites for functionalization, has become a popular tool for generation of dual function interfaces.

**Fig. 3:**
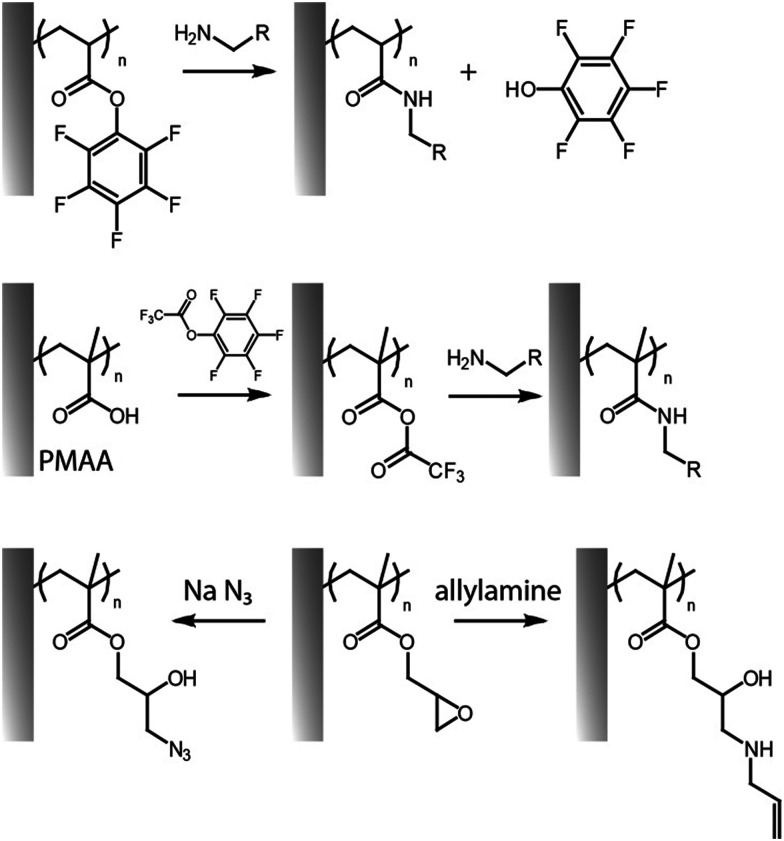
Functionalisation of pentafluorophenyl ester-activated brushes (top, reactive polymer; middle, for activation of carboxylic acid residues) and PGMA brushes (bottom).

An alternative way to couple amines to polymer brushes is by direct functionalization of reactive monomers, in particular displaying pentafluorophenyl^32,59,60,80^ and succinimidyl^31^ functions. Although similar leaving group chemistry has been applied to the activation and post-polymerisation functionalisation of PAA and PMAA,^55^ pentafluorophenyl acrylate can readily be polymerised *via* surface initiated controlled radical polymerisation, prior to direct functionalisation without requiring any activation with coupling agents (Fig. 3).^32,59,80^ Poly(pentafluorophenyl acrylate) polymer brushes (PPFA) enabled the coupling of antibodies for protein purification systems,^32^ spiropyran-amine (a photochromic compound)^60,61^ and 5-((2-aminoethyl)amino)naphthalene-1-sulfonic acid,^59^ to build light responsive and patterned fluorescent brush surfaces. One disadvantage of this approach is the relatively high hydrophobicity of pentafluorophenyl groups, but these can be hydrolysed, post coupling. Alternatively, solubility can be enhanced by partial substitution of pentafluorophenyl esters with hydrophilic moieties. For example, pre-functionalization of PPFA brushes with amino-terminated poly(ethylene glycol) (PEG) chains (10% of substitution relative to the total number of side chains) led to higher immobilization of anti-PKR antibody, presumably, as a result of more swollen chains displaying more accessible sites for coupling.^32^

Reactive polymer brushes containing NHS-esters have also been reported. For example, poly(diethylene glycol methyl ether methacrylate)-*b*-poly(2-(*N*-succinimidyl carboxyoxy)ethyl methacrylate) (PDEGMA–PSCEMA) and poly(di(ethylene glycol) methyl ether methacrylate)-*b*-poly(*N*-(methacryloxy)succinimide) (PDEGMA–PNHSMA) were used to couple 4-(trifluoromethyl)benzylamine (TFBA) for biotin coupling and streptavidin capture.^31^ In comparison, PDEGMA–PSCEMA displayed a higher degree of functionalization (around 80%) compared to PDEGMA–PNHSMA (around 5%) due to the higher reactivity and stability of the carbonate reactive ester of PSCEMA. In addition, the composition of the polymer brush with respect to the reactive monomer can be used to control the degree of functionalization. When the reactive monomer composition was varied from 80/20 to 40/60 DEGMEMA/SCEMA, the degree of functionalisation increased by 4%, which resulted in increased streptavidin immobilization on resulting biotinylated brushes.^81^

Poly(maleic anhydride-*alt*-styrene) brushes have also been proposed for direct functionalisation with various amines,^66^ although this restricts monomer compositions significantly owing to the limited polymerisability of maleic anhydride with a broad range of acrylates and methacrylates. Furan-containing copolymer brushes, such as POEGMA-*co*-poly(furfuryl methacrylate) have been successfully synthesised under mild and catalyst-free conditions. Since furan acts as an electron-rich diene, it can allow the coupling of maleimides (electron-deficient dienophiles) *via* Diels–Alder cycloaddition, which enabled functionalization with BODIPY-moieties (with a 4,4-difluoro-4-bora-3*a*,4*a*-diaza-*s*-indacene flourescent dye presenting a maleimide), and biotin for streptavidin immobilization.^82^ The degree of functionalization displayed by this approach (∼50% to 60%) was presumably limited by the bulkiness of the furan moieties. However, the regeneration capacity of the Diels–Alder products *via* retro Diels–Alder reaction (triggered at high temperatures) and further re-functionalisation was successfully demonstrated. Similarly, thiolated BODIPY and biotinylated hexa(ethylene glycol)-undecanethiol were conjugated to maleimide-containing PDEGMA brushes *via* Michael addition reaction.^83^

Finally, azide-containing monomers have also been of interest for the post-polymerization modification of polymer brushes. The azide side chains of copolymer brushes of poly(dimethyl aminoethyl methacrylate) (PDMAEMA) and poly(3-azido-2-hydroxypropyl methacrylate) P(AzHPMA) have enabled coupling of *S*-propargyl thioacetate *via* copper(i)-catalyzed azide–alkyne cycloaddition click reaction. After deprotection with sodium thiomethoxide, this allowed the formation of thiols enabling reversible crosslinking of polymer brushes *via* disulfide bonds, however, the efficiency of the crosslinking diminished over prolonged oxidation/reduction cycles due to oxidation of the thiol groups.^35^

### Activation of hydroxyl functions

Hydroxyl groups are attractive reactive groups for coupling of a wide variety of chemistries, such as carbonates and isocyanates. Isocyanate functionalized polymer brushes allow rapid and selective coupling of reactive groups such as amines and alcohols, forming urethanes and carbamates, respectively, under mild reaction conditions. For example, ring opening of epoxy groups in PGMA brushes, with sodium azide, generated free hydroxyl groups to which nitrobenzene moieties were coupled using 4-nitrophenyl isocyanate, with full conversion.^63^ The coupling of amines to disuccinimidyl carbonate-activated PHEMA and POEGMA brushes has also been widely explored, with relatively high yields.^67,69^

Another strategy proposed to exploit the high hydroxyl group density of brushes such as PHEMA brushes consists in coupling acyl chlorides and chlorosilanes to such brushes.^84–86^ These reactions are typically carried out in aprotic solvents such as dichloromethane, with chlorosilane reagents, in the presence of a base such as triethylamine. This allowed the introduction of hydrosilane groups that enabled bonding to poly(dimethylsiloxane) resins, *via* platinum catalysis. Similarly, functionalisation of PHEMA was achieved with oxalyl chloride activated triclosan (to confer antibacterial properties).^85^ An alternative approach proposed was the activation of sulfobetain residues in poly(sulfobetain methacrylate) brushes, using thionyl chloride, followed by reaction directly with triclosan,^86^ although this is likely an aggressive treatment for the brush.

Finally, hydroxyl groups can readily react with acetyl groups *via* etherification reactions catalyzed by boron trifluoride-ether complex (BF_3_–Et_2_O). For instance, POEGMA brushes grafted from a gold surface were side-chain functionalized with β-d-maltose octoacetate with formation of glycosidic bonds for glycocalyx mimicking.^64^ Interestingly, further extension of the chains was carried out *via* deacetylation of β-d-maltose octoacetate and addition of monomeric units catalysed by dextransucrase, aiming to generate a branched architecture. This outperformed the linear structures in terms of adsorption of concanavalin A.

### Coupling *via* nucleophilic substitution or addition

Nucleophilic substitutions have been broadly used for the post-functionalisation of polymer brushes, exploiting the inherent reactivity of relatively common monomers such as dimethylaminoethyl methacrylate (DMAEMA) and other tertiary amine monomers, or that of glycidyl methacrylate (GMA). Indeed, several amino monomers have been quaternized, mainly for the purpose of antibacterial coating development, using alkyl halides of various length, including methyl iodide^87,88^ and bromo undecanol,^89^ and also larger moieties such as carboxylic acids.^90^ PDMAEMA has been particularly targeted for such functionalisation as the short methyl substituents of its tertiary amine do not sterically reduce its potential for nucleophilic substitutions. The size of the alkyl chain to be grafted was also found to be important in controlling the efficiency of grafting, with methyl iodide producing close to quantitative coupling, whereas longer alkyls, such as octadecyl, typically lead to more limited efficiencies.^22,91^ Similarly, β-propiolactone was used to introduce carboxylic acids in the tertiary amine of the PDMAEMA segment, forming PDMAEMA-*co*-poly(carboxybetaine methacrylate) brushes.^90^ The degree of quaternization was readily tuned by adjusting the concentration of propiolactone and the reaction time, achieving 60% to 100% functionalization. Since subsequent adsorption of proteins was dictated by electrostatic interactions with quaternised PDMAEMA, this process was modulated by varying the copolymerisation ratios with neutral monomers. A similar approached was used to introduce charge shifting groups in the side chains of PDMAEMA brushes, using 1-acetoxyethyl-2-bromoacetate.^92^ Upon hydrolysis of the acetoxyethyl functions, carboxylic acids were generated, producing carboxybetaine residues, leading to the release of captured oligonucleotides from polymer brush-based gene delivery vectors. In most cases, nucleophilic substitutions produce changes in the brush architecture, particularly, in the measured thickness, as a result in changes in electrostatic repulsion and steric hindrance.^88,90,93,94^ Phosphorylation was another strategy applied to convert ω-hydroxyalkyl functionalised PDMAEMA brushes into zwitterionic phosphatase responsive surfaces, using polyphosphoric acid.^89^ The resulting zwitterionic polymer brushes were able to transit back to a polycationic (antimicrobial) state upon interaction with bacterial phosphatase.

Similarly, PGMA can readily be modified with amines and other nucleophiles, including thiols, azides and carboxylates.^63^ This allows grafting of a range of other moieties to associated polymer brush backbones, including radical reactive moieties, such as allylamine and propargylamine. The former was applied to thiol–ene coupling,^67^ whereas the latter could be used for reaction with alkynes through alkyne–azide Huisgen cycloaddition (see below). Epoxy groups in PGMA were shown to directly enable enzyme immobilization *via* primary amines (presumably predominantly from lysine residues), without impacting significantly on enzymatic activity, for example from glucose oxidase^95^ and laccase.^96^ In addition, the generation of cyclic carbonate groups from epoxy residues can be carried out in the presence of carbon dioxide and lithium bromide as catalyst. This was applied to PGMA brushes grafted from polystyrene-divinyl benzene microspheres to enable the covalent immobilization of laccase. Interestingly, the resulting carbonates were found to provide higher immobilization compared to epoxides (14.3 *vs.* 47.8 mg g^−1^ at 4 °C).^96^ Beyond the presence of hydrophilic polymer brushes to open the structure for diffusion, another synergistic effect has also been observed with mixed copolymers of poly(2-(diethylamino)ethyl methacrylate) (PDEAEMA) and PGMA, in which tertiary amines from DEAEMA quantitatively catalysed the ring-opening of the epoxide groups allowing enhanced amine coupling in aqueous media at room temperature.^29^ This was observed even without the incorporation of triethylamine, typically used as catalyst, in the polymerisation solution. Highest epoxide coupling to primary amines (about 48–79%) occurred when 25% of PDEAEMA was included in the composition, and gradually decreased as the PDEAEMA content decreased.

The opening of epoxide groups of PGMA can be also exploited to confer dual functionalities. For instance, ring opening of epoxides in PGMA brushes with sodium azide allowed conversion into azide (with an 85% of conversion), whilst generating alcohol groups as side chains, enabling the coupling of ferrocene *via* cycloaddition.^63^ Nucleophilic ring opening of epoxides with sodium disulfide has also been exploited for generating crosslinked PGMA brushes in a reaction that proceeds at full conversion within 5 min. The platform enabled controlled disruption of these crosslinks, *via* reduction reaction with dithiothreitol (DTT), resulting in the formation of thiol residues.^62^ Ring opening of epoxides and covalent binding to amines can also spontaneously proceed under spray-dry conditions, without the need of any additional chemical catalyst or solvent. This enabled the direct printing of antibody microarrays onto POEGMA-*co*-GMA brushes. Such printing of antibodies on reactive brushes delivered a highly sensitive immunoassay.^97^

Likewise, thiolactones can undergo nucleophilic ring opening with primary amines, forming amide bond and generating a thiol moieties. This therefore also enables dual functionalisation strategies to be developed. For example, poly(acrylamide-homocysteine thiolactone) brushes obtained *via* microwave-assisted surface-initiated radical polymerization with bromobenzyl amine^98^ were converted with modest efficiencies (54%), followed by thiol–ene Michael addition reaction of 1*H*,1*H*-perfluoro-*N*-decyl acrylate (PDFA).

### Alkyne–azide cycloaddition and thiol–ene radical coupling

**Fig. 4:**
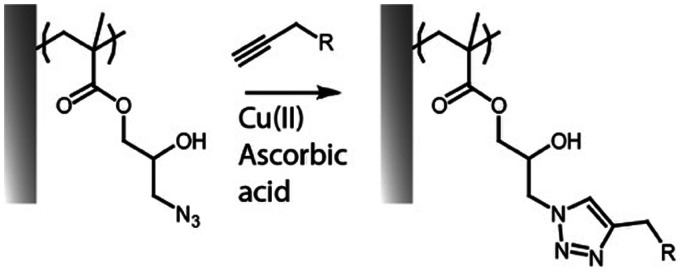
Coupling of alkynes on azide-functionalised PGMA brushes.

The cycloaddition between alkynes and azides (Huisgen cycloaddition) has been applied to functionalize polymer brushes, both through side and end chains (Fig. 4).^35,63,81^ This reaction is a 1,3-dipolar cycloaddition and results in the formation of 1,2,3-triazole cyclic adducts. Whilst non-strained alkynes require catalysis by copper(i) complexes, strained alkynes, such as those present in cyclo-octyne, can proceed rapidly, without any catalysis, enabling to broaden the range of applications of these reactions. Indeed, the metal complex used may not be compatible with some applications, in particular with biological systems, or when metal catalysts may impact on photo-physical properties. However, these strained alkynes are relatively bulky and hydrophobic, which may affect the physico-chemical properties of the brush.

While the use of copper as a catalyst has become popular to increase the conversion rate, between 10 to 100-fold, in the presence of oxygen copper can form toxic reactive oxygen species, which could limit its application in biological systems. The use of ligands, such as *N*′′,*N*′′-pentamethyldiethylenetriamine (PMDETA)^81^ can protect copper from oxidation.^99^ The incorporation of an azide group into the brush chemistry is typically achieved by nucleophilic substitution, for example, on an epoxide,^63^ as discussed previously. However, the polymerization of azide-containing monomers has also gained popularity.^35^ In addition, polymer brushes generated by ATRP present a halide end function, prone to undergo nucleophilic substitution with azides.^81^

Poly(methyl methacrylate) (PMMA) bushes generated *via* ATRP bearing bromine ends were functionalized with azides for click coupling of an alkynylated fluorescein derivative (FAM alkyne, 5-isomer) to study their conformational behaviour and change in a variety of solvents.^81^ The swollen conformation in good solvents and collapse in unfavourable solvents led to responsive fluorescence behaviours. Ensuring a tight control of the polymerisation is crucial, in order to ensure fidelity of bromine ends and high functional densities.^100^ In contrast, if termination of the living chains is desired, nucleophilic substitution of the halide end can be performed, for example, with NCB as a capping agent.^101^ In another example, PDEGMA brushes were functionalized with azides for coupling of BODIPY-alkyne dyes *via* Huisgen 1,3-dipolar cycloaddition.^83^

**Fig. 5:**
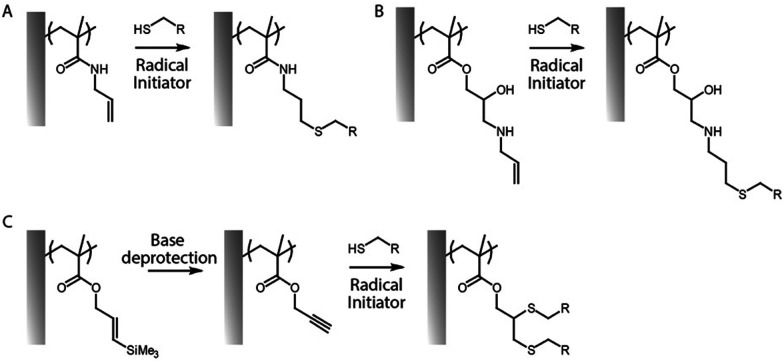
(A and B) Two examples of thiol–ene coupling applied to allylamine post-polymerisation functionalised polymer brushes. (C) Example of thiol–yne coupling to poly(propargyl methacrylate) brushes.

Thiol–ene coupling has received increased attention for the modification of polymer brushes, owing to it success for chemical design of materials in a broad range of fields (Fig. 5).^102–104^ The ability to photo-activate thiol–ene chemistry is particularly attractive in order to promote coupling in mild conditions, at room temperature, without requiring oxygen protection. Hence, a range of different thiols, can readily be coupled to polymer brushes decorated with alkene residues, typically introduced post-polymerisation, for example *via* coupling of allylamine or propargylamine to PGMA^67^ or poly(pentafluorophenyl methacrylate) (PPFMA).^105^ Functionalisation levels remained modest, ranging between 30 to 80%, depending on the thiol and the type of alkene/alkyne used.^67^ However, kinetics of functionalisation were found to be fast (reaching plateaus within 2–5 min) of photo-irradiation. In addition, the ratio of thiol to photoinitiator used was found to be essential to enhance coupling efficiencies, with improved coupling at high thiol/initiator ratios, presumably due to improved transfer to thiols and persistence of radicals under the conditions tested.^67^ Indeed, in the absence of thiols, initiators were found to directly couple to the brush, potentially leading to rapid loss of radicals for further coupling.

In contrast to azide–alkyne cycloadditions and thiol–ene coupling reactions, the thiol–yne reaction allows the addition of two thiol molecules for each reactive (alkyne) site.^68^ Therefore double hydrothiolation of alkyne groups allows higher densities of thiols to be achieved, potentially increasing the functionality of surfaces.^106^ An alternative strategy based on thiol–ene coupling was to thiolate polymer brushes *via* the reaction of cystamine with poly(maleic anhydride-*co*-styrene) brushes, followed by reaction with various acrylates.^107^ This approach has the benefit of enabling access to a broad range of acrylate building blocks for functionalisation, although possible disulfide bond formation may affect the brush swelling and physico-chemistry.

In order to bypass the need for post-polymerisation modification, azido monomers such as 3-azidopropyl methacrylate can be directly polymerised prior to cycloaddition. These brushes were found to grow well, even to thicknesses above 100 nm.^108^ Interestingly, copolymers with POEGMA displayed increasing hydrophilicity with increasing fraction of oligo(ethylene glycol) side chains, which improved the efficiency of post-polymerisation cycloaddition. This allowed the coupling of alkyne-bearing dyes, as proof of concept. Similarly, alkenes were introduced as side chains of oligo(2-alkyl-2-oxazoline)methacrylates, using the opportunity of synthesis of oligo(2-alkyl-2-oxazolines) with telechelic functional groups (*e.g.* one ATRP active methacrylate moiety and one unreactive allyl residue).^109^ This led to moderate thiol–ene coupling, using photo-activation, depending on the brush thickness and irradiation time. An alternative approach consists in polymerising protected alkene or alkyne functions, for example using silane-protected alkynes (Fig. 5)^66^ or furan-protected maleimides.^110^ After surface-initiated polymerisation of corresponding methacrylates, the reactive alkynes and maleimides can be deprotected by treatment with alkaline solutions or *via* retro-Diels–Alder reaction, respectively, prior to functionalisation with thiols.

### Other strategies proposed for side chain functionalisation

A few additional strategies explored for the functionalisation of the side chain of brushes have been proposed. These aim to achieve functionalisation in mild conditions, for example compatible with protein coupling, or upon photo-irradiation. For example, aldehyde-functionalised polymer brushes were generated using an aldehyde-terminated pentaethylene glycol methacrylate.^70,111^ To simplify the synthesis and polymerisation, these brushes were also generated by polymerising a propylene glycol terminated monomer, prior to selective oxidation of the diol in mild condition (sodium periodate), to form the corresponding aldehyde. Subsequently, these aldehyde functions were applied to the functionalisation of brushes with histidine residues, directly *via* Schiff base chemistry, resulting in responsive polycationic brushes. This approach is attractive as not introducing bulky or hydrophobic residues on the brush structure.

In order to enhance the adhesion of brushes to various biological substrates, including tissues, diazirine chemistry was proposed for the tethering of polymer brush functionalised substrates.^14^ In this approach, the high functional density of PHEMA brushes was used to introduce 4-[3-(trifluoromethyl)-3*H*-diazirin-3-yl]benzoic acid residues (*via* carbodiimide coupling). Upon photoirradiation, diazirin residues degraded into carbene moieties that could then couple to the target tissue (bovine meniscus in this case), promoting strong adhesion of the substrate or implant of interest. Another strategy introduced to promote photo-activated coupling to brush side chains is the nitrile imine-mediated tetrazole–ene cyclocloaddition.^71^ Functionalisation of PHEMA brushes with tetrazole moieties (using acyl chlorides, in anhydrous conditions) was followed by photoactivation in the presence of maleimides that enabled the tethering of associated molecules such as biotin or ATRP initiators (for subsequent further polymerisation). Although relatively long exposure times were required, this approach enabled the patterning of the brush chemistry.

### Impact of the brush architecture

The localisation of the functionalities introduced through the various coupling strategies discussed so far is typically assumed to be homogenous throughout the brush. However, it is possible, and indeed supported by evidence, that functions are localised in specific compartments of the brush, depending on the brush architecture and the chemistry of the moieties introduced. Resolving the spatial localisation of chemical functions within brushes, at the nanometer scale, remains challenging and relies on the combination of techniques. This is often incompletely understood, but the *z*-localisation (in the direction perpendicular to the plane of the substrate) has been investigated in a number of cases.

**Fig. 6:**
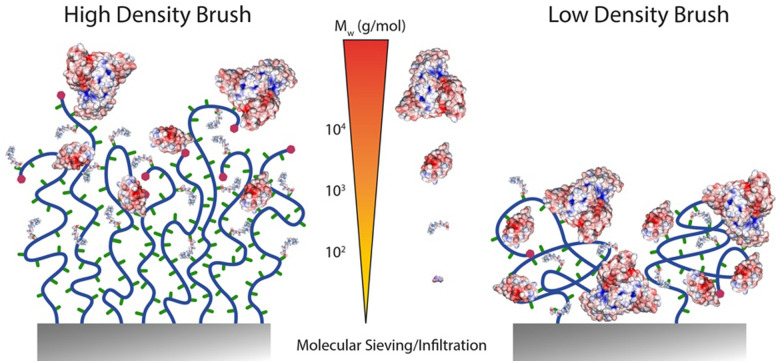
Schematic representation of expected functionalisation density profiles depending on the molecular weight of molecules to be coupled to dense and sparse polymer brushes.

Typically, two relatively straight forward strategies to characterise functionalisation levels include FTIR spectroscopy and ellipsometry. Whilst FTIR can be quantitative, providing suitable internal reference is accessible, the use of ellipsometry to quantify functionalisation levels requires to make assumptions regarding potential changing in the density of polymer brushes, upon functionalisation.^22,67,112,113^ However, both techniques typically average signals over the full thickness of the brush and offer relatively low lateral spatial resolution. Results obtained from such techniques can be contrasted with XPS data, which are associated with a more restricted *z*-profiling, within the top 5–10 nm of the brush. Hence, there is evidence established with a range of different polymer brushes that small molecules can diffuse and functionalise lateral chains throughout polymer brush coatings, whereas larger molecules are restricted to the upper layers of the brush^22,67^ (Fig. 6).

To gain further insight into the *z*-profile of the brush chemistry, neutron reflectometry and X-ray photoelectron spectroscopy with depth profiling have been applied. Neutron reflectometry, being particularly sensitive to changes in scattering length density perpendicular to the plane of reflection, was used to establish that the size of amines coupled to PHEMA brushes activated with nitrophenyl chloroformate (NPC) impacted the localisation of functionalised moieties.^114^ Hence, whereas deuterated serine coupled relatively homogenously throughout dense brushes (not measured in this study, but based on other studies using comparable surface-initiated polymerisation protocols, likely in the range of 0.5 chains nm^−2^), D10-leucine functionalisation was restricted to the surface of the brush. The thickness of functionalised brushes was remarkably insensitive to the total thickness of the brush, further confirming that limitation of diffusion is likely accounting for the behaviour observed. In contrast, both amines investigated functionalised throughout NPC-activated PHEMA brushes with reduced grafting densities. These results are comparable to XPS data with depth profiling, which confirmed the functionalisation of PGMA brushes with propylamine, whereas large macromolecules were restricted to the very top surface of the brush,^115^ although such effect may also be associated with the hydrophobicity of PGMA brushes and their potential crosslinking, leading to limited infiltration.

## End chain functionalisation strategies

Whilst polymer brushes offer opportunities for high chemical function densities at interfaces to be achieved, in some applications, only functions present in the upper layers of the brush are effective or necessary. This is the case of interfaces designed to promote cell adhesion or for biosensing, where large macromolecules, receptors or cells might not be able to penetrate deeply within the brush structure.^116,117^ Therefore, strategies to end-functionalise polymer brushes are attractive to confer structure and to confine chemical functionality at the apical surface of polymer brushes (Table 2 and Fig. 1).

**Table 2:** Examples of end-chain functionalisation strategies applied to polymer brush design

Brush type	CRP	Architecture	Post-polymerization modification required	Molecule(s) coupled	DF	Application	Ref.
PHEMA	ATRP	*h* = 56 ± 0.1 nm	Nucleophilic substitution with amine residues	Gelatin	n.s.	Cell culture	37
PDMAA–PNHSA		*σ* = 0.6 chain nm^−2^					
PHPAA–PNHSA							
PNAM–PNHSA	SET-LRP	*h* = <10 nm	Amide bond formation triggered by NHS-ester	c(RGDfK) and c(RADfK) peptide	n.s.	Cell adhesion	119
PMMA	ATRP	*h* = 25 nm	Copper-catalysed alkyne–azide cycloaddition	Alkynylated fluorescein	n.s.	n.s.	81
		*σ* = 1.13 chain nm^−2^					
PDEGMA	RAFT	*h* = 52 ± 2 nm	Azobis–azide, azobis–maleimide and azobis–alkenes for azide–alkyne, thiol–maleimide, and radical thiol–ene couplings	Fluorescent dyes, biotin and mannose	n.s.	n.s.	83
		*σ* = 0.64					
		chain nm^−2^					
POEGMA	ATRP	*h* = 33 ± 0.6 nm	Nucleophilic substitution with NaN_3_	Biotin and streptavidin	n.s.	Antifouling surfaces	137
			Strain-promoted alkyne–azide cycloaddition				
POEGMA	ATRP	*h* = 20 nm	Nucleophilic substitution with NaN_3_	Biotin, streptavidin and biotinylated antibody	n.s.	Biosensing	121
			Strain-promoted alkyne–azide cycloaddition			Antifouling surfaces	
POEGMA	ATRP	*h* = 10–30 nm	Nucleophilic substitution of pentadiene to alkyl halide for further Diels–Alder “click” reaction.	bis(cyclopentadienyl) nickel(ii) (nickelocene, NiCp2) for coupling of maleimide-BSA	12–67%	Antifouling surfaces	118
PBMA	RCMP	*h* = 12 nm	Hetero-disulfide exchange	Thiolated dye, end-thiolated polymer	>78%	Rewritable surfaces	120
PHEMA							
POEGMA	ATRP	*h* = 30 nm	Carboxylation and amide bond formation *via* carboxylic acid activation to carbonate-ester	*N*,*N*′-Disuccinimidyl carbonate for coupling of *N*,*N*-bis(carboxymethyl)-l-lysine (NTA) and immobilization of His-tagged green fluorescent protein	n.s.	Antifouling surfaces	124
PNIPAM	LRP	*σ* = 1.61 chain nm^−2^	Nucleophilic substitution	Cysteamine	n.s.	n.s.	
PNIPAM–PGMA	ATRP	*σ* = 0.7 chain nm^−2^	Nucleophilic with amino-alkyne for click reaction	Propargylamine	72%	n.s.	139
PS	ATRP	*h* = 6–7 nm	Nucleophilic azidation	Alkynes *via* click cycloaddition	85%		100
PMMA		*σ* = 0.42–0.47 chain nm^−2^			95%	n.s.	
PNIPAM	SET-LRP	n.s.	Nucleophilic azidation	Propargylated RAFT agent	n.s.	Block copolymer brush growth	140
PGMA	RAFT	*h* = ∼16 nm	EDC/NHS from carboxylated RAFT agent	RGD peptides	n.s.	Cell adhesion	141
Polymethylene	Polyhomologation	*h* = 25.0 ± 1.8 nm	Oxidation of boronic esters to alcohols	Polycaprolactone block	n.s.	Chain extension	142
		*σ* = 0.57 ± 0.02 chain nm^−2^					

The open linear structure of polymer brushes is well-suited for end-chain functionalization, allowing the preservation of the chemical properties of the brush backbone, with minimal impact over its architecture.^81,118^ Although functional densities achieved *via* end-chain functionalisation are inherently limited by the density of brushes, even when near quantitative coupling is achieved, highly specific binding capacities can be exploited for biosensing applications, especially when the activity of the coupled molecules is affected by their orientation/confinement (*i.e.* for antibody or receptor ligand immobilisation).^32,44^ While it is reasonable to assume that end-functionalisation may be facilitated, due to the more exposed nature of associated reactive end groups, the degree of functionalization and immobilization of large molecules can be affected by the bulkiness of the moieties and associated steric hinderance,^119–121^ as occurs in side-chain functionalisation.^29,82^ However, in many cases, brush densities achieved *via* controlled radical polymerisations remain particularly high, compared to the densities of functions required by relevant applications. For example, cell adhesion is sensitive to ligand densities with spacing above 60 nm spacing and many biomarkers form dense monolayers restricted by the dimensions of proteins (often above 3 nm).^122–124^ Hence, these densities (within a range of 3 × 10^−4^–0.1 molecules nm^−2^) are comparable or several orders of magnitude lower than the densities of polymer brushes achieved by a grafting from approaches (typically 0.1–0.5 chains nm^−2^).

However, achieving relevant surface densities requires relatively high coupling efficiencies for end chain functionalisation at reasonably low reactant concentrations. Click reactions, thermodynamically-favoured reactions leading almost exclusively to one product, proceeding quantitatively under mild conditions even in dilute systems, including in biologically relevant conditions, are attractive candidates for end-chain functionalisation of brushes.^125,126^ This is particularly important for biofunctionalization, where the amount of ligands available for coupling is intrinsically limited by their production cost and difficulty to scale up, as well as their chemical stability.^126^

**Fig. 7:**
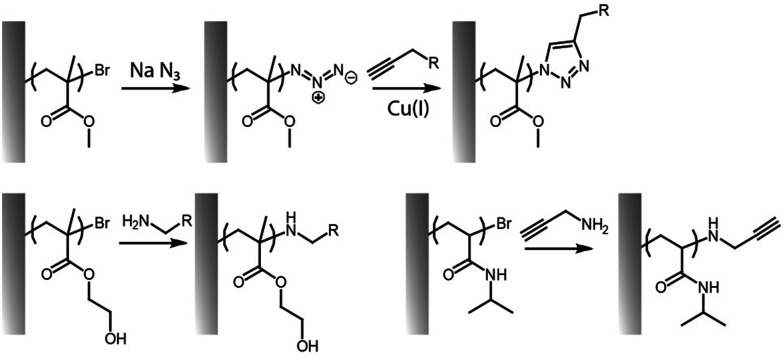
Examples of end-chain functionalisation of polymer brushes, based on nucleophilic substitution reactions to convert bromide end groups to reactive alkynes or azides for subsequent click reactions.

Several click reactions have been reported for the functionalization of polymer brushes, among them cycloadditions, such as Diels–Alder and alkyne–azide reactions, and thiol–ene couplings, including Michael additions and radical thiol–alkene and thiol–alkyne reactions remain the most widely applied.^103,104,127,128^ However, other emerging highly efficient reactions, such as sulfur(vi) fluoride exchange chemistry (SuFEx)^129–131^ and hetero-disulfide exchange reactions^120,132,133^ have received some attention for side-chain and end-chain functionalisation of brushes. Associated with this diversity, some of the members of click chemistries are orthogonal (*e.g.* alkyne–azide cycloaddition and thiol–ene coupling) and can be carried out in parallel or sequentially, without requiring much purification or processing.^23^ These coupling strategies are also compatible with the inherent chemistry of end chains associated with ATRP or RAFT processes, presenting bromide or trithiocarbonate groups that can be converted into azide, alkyne, thiols or alkene residues conveniently (Fig. 7). For example, trithiocarbonates can be converted into thioethers with various functionalities, including cations,^134^ whereas carboxylic groups of trithiocarbonate initiators were used for subsequent amide bond formation.^135^ End-chain dithioesters resulting from RAFT polymerisations were also shown to allow coupling with azobis derivatives presenting azide, furan-protected maleimide and terminal alkene moieties.^136^

Cycloadditions between dienes (such as cyclopentadiene or furan) and dienophiles (such as maleimide) are known as Diels–Alder reactions.^82^ To proceed, the diene should be electron rich, whereas the dienophile should display an electron deficient character. These reactions are typically thermo-reversible, enabling formation of the adduct at low temperatures and their reversal upon heating (entropically driven).

Several examples of this reaction have been presented for both side-chain^82^ and end-chain^118^ functionalization of polymer brushes. Recently, ultra-fast and metal free Diels Alder reaction was used to functionalize POEGMA brushes with cyclopentadienyl moieties, to undergo click coupling with maleimide-BSA.^118^ The degree of click-functionalization varied as a function of the brush thickness, namely, 67% (838 pg mm^−2^), 25% (314 pg mm^−2^) and 12% (154 pg mm^−2^), for thicknesses of 10, 20, and 30 nm. This trend presumably reflected the gradual loss of end group fidelity with increasing polymerisation times. Poly(styrene) (PS) brushes displayed the same tendency with lower end-chain azide fucntionalization with thicker brushes, but this was not the case for PMMA brushes, where the thickness was not found to influence the ultimate end-chain conversion.^100^

**Fig. 8:**
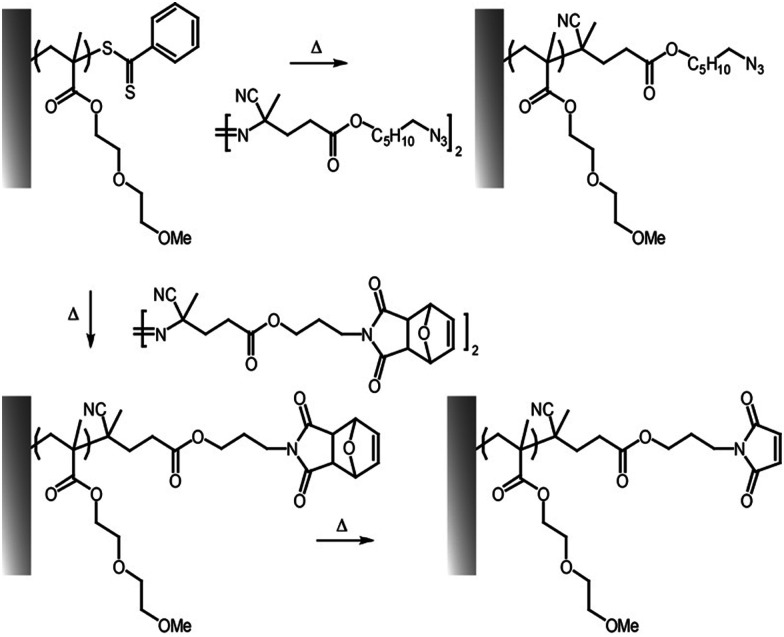
Examples of end chain functionalisation of polymer brushes based on the coupling of azobis-derivatives to introduce azide and maleimide residues (*via* retro-Diels–Alder reaction).

The cycloaddition of an azide and alkyne has also broadly been applied to the end-chain functionalisation of polymer brushes, making use of the simplicity of conversion of halides to azides (although approaches to develop azide or alkyne-end functionalised brushes from RAFT have also been proposed). Whilst non-strained alkynes require catalysis by copper(i) complexes, strained alkynes, such as those present in cyclo-octyne, can proceed rapidly, without any catalysis, enabling to broaden the range of applications of these reactions. Indeed, the metal complex used may not be compatible with some applications, in particular with biological systems, or when metal catalyst may impact on photo-physical properties. However, these strained alkynes are relatively bulky and hydrophobic, which may affect the physico-chemical properties of the brush. This strategy has nonetheless been applied to the functionalisation of POEGMA,^118,121,137^ PDEGMA,^83^ PNIPAM,^140^ PMMA^81,100^ and PS^100^ brushes end-terminated with azido functions, enabling a simple and efficient route to tether functional molecules, from dyes to biotin residues (Fig. 8).

The reaction of a nucleophile (typically a thiol or amine) with an electron deficient activated alkene is known as a Michael addition. The mechanism involves a carbanion intermediate that is stabilised by electron withdrawing groups. Hence typical alkenes used include maleimides, acrylates and vinyl sulfones.^143^ Nucleophiles routinely used are also typically involved in acid–base equilibria and therefore moderately alkaline pH (typically 8.0–9.0) are required to deprotonate these reagents, in order to increase reaction rates.^144^ Thiols are also typically more reactive, due to the higher polarizability of the sulphur atoms.

In contrast to Michael additions, radical-based thiol–ene coupling is specific to thiol residues, as thiyl radicals are essential intermediates.^103^ Therefore, the pH of the reaction should be neutral to modestly acidic in order to ensure that sufficient concentrations of thiols (with p*K*_a_ typically in the range of 7.0–9.0) are protonated and can form thiyl species upon radical initiation.^145^

Thiols and maleimide functions can be conveniently introduced at the end of polymer chains generated *via* RAFT or ATRP, for example through nucleophilic substitution with amines^37,138,139^ and dithiols of heterdifunctional thiol–disulfides.^120^ Hence, end-thiolated polymer brushes enabled the subsequent coupling of maleimides. This was exploited to form hetero-disulfides and further coupling with thiolated polymer (fluorinated, in this case), and to couple maleimide-functionalised dyes, creating fluorescent patterns.^120^ Similarly, alkene-terminated polymer brushes, whether based on maleimide or terminal alkene residues, enable the couple of thiols, confining fluorescence (*e.g.* BODIPY or rhodamine dyes) or specific recognition (*e.g.* biotin) to the upper surface of the brush.^83^

## Chemical patterning and structuring of polymer brushes

The rapid development of micro- and nano-fabrication techniques have paved the way to the engineering of structured interfaces for a broad range of applications, from electronic chip and MEMS to sensors and cell-based assays.^4,6–9,146^ Polymer brushes have been broadly applied to these technologies and offer unique opportunities for precise nano-to-micro-scale engineering of the chemistry of interfaces. This section of our perspective article will focus on recent reports allowing the nano- and micro-structuring of the brush chemistry, briefly reviewing key patterning approaches allowing the spatial structuring of polymer brushes, prior to presenting strategies to directly pattern homogenous reactive brushes with chemical and biochemical functionalities. The third focus of this section is the *z*-structuring at the nanoscale (perpendicular to the plane of the substrate).

### Chemical patterning of interfaces using polymer brushes

A broad range of strategies have been proposed for the patterning of polymer brushes. Perhaps the most broadly applied have been the microcontact printing of initiators on gold substrates,^17,147–150^ the patterning of brushes using photoresists^151–153^ or using alternative masks to prevent brush growth, as in electrospun nanofibre lithography.^154,155^ Other methods of initiator deposition have included the inkjet printing of macroinitiators, resulting in scalable sub-micron resolution brush patterns.^156^ These approaches typically display resolutions in the micrometer scale and enable the patterning of a broad range of polymer brushes, as the initiator used for controlled radical polymerisations can enable the introduction of a wide range of monomers.

More recently, a range of approaches have been introduced to directly pattern initiators for controlled radical polymerisations, for example through the photo-degradation of initiator monolayers using deep UV irradiation.^157^ This has the advantage of relying on established chemistries and initiator monolayers, and enables in principle high resolution as the wavelength of the light used is lower than that typically used for photolithography. For example, silane initiators bearing chloromethyl benzyl residues were irradiated with deep UV (244 nm), resulting in their oxidation to corresponding carboxylic acids. This was followed by ATRP. Interestingly, residual groups could be converted to new ATRP initiators to generate patterned binary brushes.^158^ Another approach recently proposed was the deposition of a polymer layer decorated with ATRP initiator moieties, *via* the chemical vapor deposition of [2.2]-paracyclophane-4-methyl 2-bromoisobutyrate.^159^ This enables the formation of an initiator layer in a substrate-independent manner. In turn, the resulting poly[(*p*-xylylene-4-methyl-2-bromoisobutyrate)-*co*-(*p*-xylylene)] films can be photo-etched using deep UV irradiation (185 to 257 nm), leading to the generation of ATRP initiator patterns remaining in protected areas. This strategy can then be used for the growth of polymer brushes, for example controlling protein fouling and patterning, or the generation of reactive brushes bearing alkyne residues for subsequent copper(i)-catalyzed azide click coupling (for example for the introduction of biotin residues for the capture of streptavidin).

Instead of triggering the degradation of initiators, another strategy recently proposed was the use of photo-irradiation to pattern the surface chemistry to enable the coupling of initiators. The assembly of *N*-[2-(2-nitrophenyl)propan-1-oxycarbonyl]-protected aminopropyl silane monolayers, followed by photo-activated deprotection allowed the patterning of bromoisobutyryl residues.^160^ The resulting ATRP initiator patterns allowed the generation of polymer brushes with μm resolution. In addition, residual nitrophenyl protected areas could be further irradiated to reveal new amines and subsequent initiator coupling. This enabled the formation of binary polymer brush patterns, for the μm-resolution confinement of supported self-assembled lipid bilayers. An analogous strategy was proposed for the patterning of brushes at the surface of polyesters. An acetal protected polyester was combined with a photoacid generator in order to trigger the formation of hydroxyl residues at the substrate surface, prior to coupling of bromoisobutyryl residues.^161^ This enabled the patterning of brushes from resulting surfaces. Another photo-triggered approach was to apply thiol–ene radical reactions to the direct patterning of nitroxide-mediated initiators, through the coupling of a thiolated alkoxamine to undecenylsilane monolayers.^162^ This approach may be applicable to ATRP initiators, to broaden the range of polymer brushes and substrates that can be targeted. An alternative approach to control the etching of thiol monolayers from gold substrate was proposed to use irradiation-promoted exchange reactions of thiols, promoted by electron beam lithography.^163^ After polymer brush growth *via* ATRP, this resulted in sub-micron patterned substrates.

The need to pattern initiators in order to microstructure polymer brushes was recently lifted by the emergence of digital mirror and light projection systems. For example, the direct patterning of brushes was achieved using a digital light projection system to induce electron transfer-reversible addition–fragmentation chain transfer (PET-RAFT) polymerisation. This was applied to the multi-component printing of structured polymer brushes. The resolution of these platforms is in the range of a few tens of μm, depending on the platform used for irradiation, but also enables greyscale patterns to be generated.^164^ A similar concept was applied to the generation of multicomponent brush patterns, with μm resolution in the plane of the substrate and nm resolution in the *z*-direction, combining an irridium-based ATRP system and a digital micromirror device.^165^ Notable is also the application of an organocatalytic controlled radical polymerisation, reversible complexation mediated polymerization, making use of iodo-initiators catalysed by the transfer agent 1′,3′-dihydro-8-methoxy-1′,3′,3′-trimethyl-6-nitrospiro[2*H*-1-benzopyran-2,2′-(2*H*)-indole] (DHMI). This approach, combined with the ability to photo-degrade iodo-initiators, enabled the formation of complex micro-structured polymer brush patterns.^166^ Alternatively, thiol initiators were activated using photo-radical generators and irradiation through elastomeric pyramidal tips (beam pen lithography), leading to polymer brush growth. To enable control of brush growth, the dwell time and the use of microfluidic parallelised chemical microreactors were applied to generate polymer patterns, with potential for combinatorial approaches.^167,168^

Finally, in order to enhance the resolution of polymer brush patterning without compromising on design flexibility and scalability, dip pen nanolithography has been broadly applied for the printing of thiol initiators for ATRP.^169^ This enabled the patterning of brushes with sub-μm resolutions (50–500 nm routinely), on relatively large scales,^170^ enabling the control of brush morphologies.^171,172^ When combined with reactive or functional polymer brush growth, DPL therefore enabled the creation of functional fluorescent and protein arrays^170^ or the control of etching of nanostructures.^173^ DPL was also applied to other polymer brush growth strategies, such as ring opening metathesis polymerisation, using immobilised ruthenium catalysts after printing of norbornenyl thiols patterns.^174^

### Chemically patterned polymer brushes

The functionalisation of patterned polymer brushes has been broadly applied to confer a range properties to these coatings, from sensing to biorecognition and cell adhesion. Many of the strategies proposed share similarities with the chemical patterning of other interfaces, such as self-assembled monolayers.^175^ The simplicity of fabrication of a range of reactive brushes, or brushes that can be activated in mild conditions, has enabled the generation of functional polymer patterns, combining some of the chemistries described in previous sections of this review with polymer brush patterning platforms discussed above.

The direct patterning of reactive polymer brushes or copolymer brushes is attractive as reducing the number of steps required for functionalisation of resulting brush arrays. For example, PET-RAFT was used for the sequential patterning of two different types of reactive polymer brushes (poly(glycidyl methacrylate)) (PGMA) and poly(2-(2-azido-2-methyl-1-oxopropoxy)ethyl methacrylate) (PAMEMA), enabling the subsequent coupling of proteins (streptavidin, to PGMA) and fluorescent dyes (using 3-dibenzocyclooctyne conjugates, reacting without copper activation to PAMEMA).^176^ Similarly, surface initiated PET-RAFT and a digital light projector were combined to create poly(dimethylacrylamide), POEGMA and poly(pentafluorophenyl methacrylate) brush patterns that could be further coupled with amines (*e.g.* Alexa Fluor 488 conjugates).^164^

More broadly, a wide range of strategies have been proposed for the functionalisation of patterned polymer brushes with various molecules and proteins. Protein resistant polymer brush patterns (based on POEGMA or zwitterionic brushes) have been widely used to create protein patterns *via* simple deposition to unprotected areas upon incubation of substrates in protein solutions.^17,18,148,177^ This strategy has the advantage of allowing the deposition of proteins at low concentrations (usually a few μg mL^−1^), without further functionalisation or activation. More recently, other approaches were proposed to create protein adhesive patterns guided by protein resistant polymer brushes. For example, the creation of a patterned polydopamine coating at the surface of the brush, enabling subsequent protein adsorption.^178^ Another strategy reported was to introduce glycidyl methacrylate groups in areas not protected by brushes, to enable covalent coupling to underlying substrates.^179^

The coupling of a range of molecules to patterned polymer brushes has been achieved. For example, thiols (including from cysteine-bearing peptides) can be coupled to patterned brushes presenting maleimide residues without further activation.^110^ Biotin residues and proteins have been tethered to side chains or end chains of polymer brushes *via* amine coupling to NHS- or DSC-activated residues, allowing the subsequent capture of streptavidin and other biotinylated proteins, to generate protein patterns.^180–183^ Another strategy recently proposed to pattern nanoparticles (silica nanoparticles and liposomes), potentially applicable to a broader range of nanostructures and macromolecules, consists in decorating adamantane residues on a brush, in order to capture cyclodextrin-functionalised nanoparticles and vesicles.^162^ In this case, nitroxide initiators were patterned prior to the growth of poly(2-adamantyltriethoxyethyl acrylate) brushes. As cyclodextrins can be readily decorated on a broad range of macromolecular structures, this approach could enable much flexibility in the design and functionalisation of brushes.

A number of strategies have also been proposed to chemically pattern homogenous polymer brushes. In these reports, a continuous homogenous polymer brush is first generated from a substrate of interest, prior to the patterning of its chemistry. thiol–ene radical coupling is attractive for such applications as alkenes and alkynes can conveniently be introduced as side chains of polymer brushes, followed by coupling of targeted thiols using photomasks to structure irradiation.^67,69^ This enabled the formation of cell adhesive peptide patterns, for example, promoting specific cell adhesion.^69^ Unreactive alkenes introduced at the end of poly(2-alkyl-oxazoline methacrylates) can also be directly polymerised *via* ATRP, followed by thiol–ene coupling of thiols and peptides activated through photomasks.^109^ Thiolated brushes (generated through coupling of cysteamine to poly(styrene-*alt*-maleic anhydride) brushes) can be functionalised with a range of alkenes.^107^ An alternative to the use of photomasks is to introduce thiols and alkenes at the surface of brushes through microfluidic channels or silicone stamps. Indeed, microchannels allow the compartmentalisation of molecules to be tethered, prior to photoactivation, although with limited resolution (as the dimension of microfluidic channels is restricted by pressures associated with injection and flow of corresponding liquids, even with low viscosity).^107^ Reactive microcontact printing, achieved by placing silicone stamps inked with thiols, in contact with an alkene-functionalised brush, allows to achieve resolutions typical of associated photolithography platforms.^67^ In turn, brushes have also been proposed to allow the transfer of small molecules such as silanes, using microcontact printing. For such application, poly(*N*-[tris(hydroxymethyl)-methyl]acrylamide) brushes were generated from the surface of silicone stamps, enabling the reversible capture of silanes that can be transferred to target substrates.^184^

Finally, the patterned crosslinking of polymer brushes has recently received some attention. For example, poly(furfuryl methacrylate) brushes can be crosslinked using bis-maleimide crosslinkers, through Diels–Alder reactions.^185^ When disulfides were introduced in the core of the crosslinkers, their reduction allowed further coupling of different maleimides or alkyne residues to generate functional patterns. Another approach recently proposed to crosslink brushes consists in introducing *o*-methylbenzaldehyde (*o*-MBA) residues as side chains of methacrylate brushes.^186^ These moieties can then be dimerised upon photoirradiation (325 nm light), effectively crosslinking the brush and impacting its swelling and mechanical properties. Finally, another notable approach that was recently reported to enable the direct writing of conductive patterned structures consisted in growing terthiophene methacrylate brushes that could be subsequently crosslinked *via* electropolymerisation.^21^ Conducting AFM was used to locally trigger such reaction, resulting in the formation of conducting nanowires.

### *Z*-Structuring the brush chemistry

**Fig. 9:**
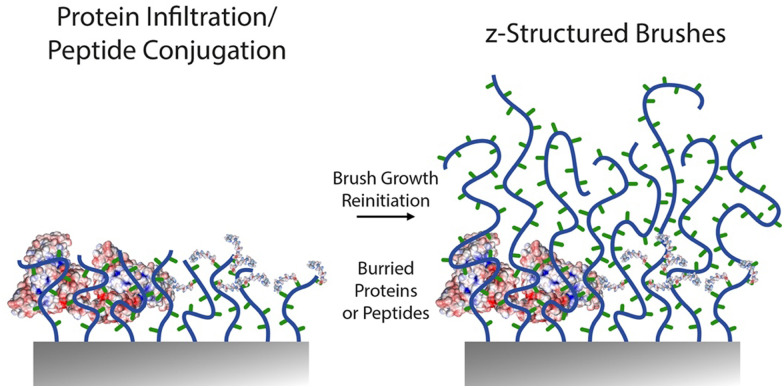
Schematic representation of recent approaches proposed for other *z*-structuring of biofunctionalised polymer brushes. A first block of brush is generated, followed by protein adsorption or peptide conjugation, prior to re-initiation of a second polymer brush block.

An important feature of polymer brushes generated *via* surface-initiated controlled radical polymerisations is the ability to re-initiate growth to generate block copolymer brush architectures.^4,146^ This has not only enabled the *z*-structuring of polymer brush topographies and morphologies,^165,176,187^ but also enabled the re-initiation of brushes to control the localisation of key chemical functions^188^ (Fig. 9). The control of end chain functionalities is also an attractive feature of ATRP, RAFT and NMP, for chemical *z*-structuring, for example relying on bromides conversion to azides and alkyne coupling,^189^ or making use of terminal thiols accessible through RAFT for end chain functionalisation or brush structuring.^190^

For example, block copolymer architectures were proposed to allow the control of specific biomarker recognition, without compromising on the protein resistance of the coating. Indeed, ultra-low fouling brushes based on zwitterionic carboxybetaine and oligo(ethylene glycol) derivatives allow the almost complete suppression of non-specific fouling from complex protein mixtures (including serum, plasma and blood),^191–193^ but their functionalisation can be difficult or even detrimental to their protein-resistance performance.^180^ A proposed strategy to address this issue was to develop block copolymer architectures presenting a lower block with high density and protein resistance and an upper block functionalisable and potentially displaying lower density. Hence, partial re-initiation of poly(carboxybetaine) brushes, followed by antibody coupling *via* EDC/NHS resulted in sensors with improved binding capacity, without compromising on specificity.^194^ Similarly, POEGMA brushes can be re-initiated with short PGMA blocks that can be converted to azides prior to coupling with strained alkynes, for example enabling biotinylation and antibody tethering.^189^ Alternatively, end chains of brushes grown *via* ATRP can directly be converted into azides, prior to cycloaddition, but this strategy was not found to enable sufficient levels of subsequent antibody capture.

The *z*-structuring of brushes was also applied in two separate studies to the investigation of the impact of integrin ligand “burying” and brush morphology on cell adhesion. In both cases, block copolymer brushes were grown, to present RGD peptide functionalised blocks from which upper unfunctional blocks were grown.^116,117^ A key difference between these two studies is the nature of the brushes used, one being a polyanionic methacrylic acid brush,^117^ whereas the other being based on neutral PHEMA.^116^ Both studies concluded that burial of ligands inhibited cell adhesion. From a functionalisation point of view, the choice to re-initiate the brush after peptide functionalisation highlights the difficulty to allow diffusion and coupling of even moderately large molecules within polymer brushes. Therefore, placing adhesive ligands at the outer layers of the brush is sufficient to promote cell adhesion and this strategy can enable promoting selective cell adhesion, for example from polyacrylamide brushes^195^ or POEGMA brushes.^105,176^ However, the physico-chemistry and morphology of lower brush compartments do modulate this behaviour and associated cell response, by impacting on nanoscale mechanics. This result is analogous to the response of cell adhesion to end-functionalised grafted-to polymer brushes, which mediated cell adhesion in a chain length dependent manner, highlighting the importance of chain displacement and associated local mechanics on the reinforcement of cell adhesions.^196^ For such functional *z*-structured coating design, EDC/NHS and thiol–ene coupling were used to achieve controlled peptide coupling.

Finally, a difficult issue to tackle with biofunctional brush design, for example for biosensing applications, is the correct positioning of proteins within the brush coating. Indeed, whereas antibodies are preferred at the outer layers of the brush to enable biomarker binding, electrochemical sensors, for example based on impedance spectroscopy, require the positioning of electroactive elements close to the brush substrate. This is typically challenging for dense polymer brushes that restrict protein infiltration and coupling. However, recent work showed the ability to reinitiate brush growth *via* ATRP following infiltration of proteins within a first short block.^197^ Therefore, controlled radical polymerisations may enable the high resolution *z*-structuring of a broad range of architectures for sensing and responsive behaviour and applications.

## Biofunctionalisation of polymer brushes

**Fig. 10:**
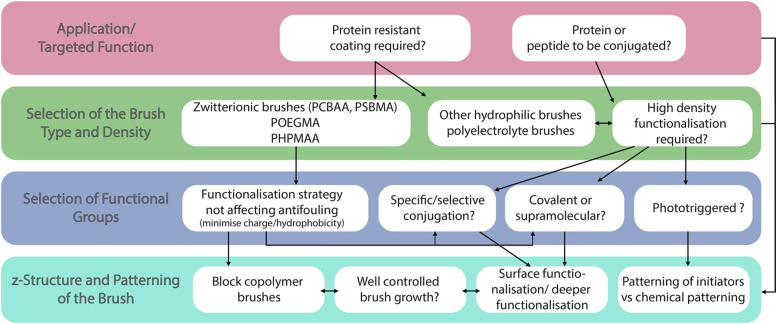
Guided decision for polymer brush biofunctionalisation.

The functionalisation of polymer brushes with peptides, amino acids and proteins has been described for a variety of applications, from antimicrobial coatings^38,39,42,43^ and purification systems/membranes^32,60^ to biosensing^31^ and cell adhesion platforms^36,37,40^ and patterning.^59^ In the field of regenerative medicine, polymer brushes are also attractive coatings to modulate biological interactions and for the design of new generation of drug delivery systems.^198,199^ Indeed brushes are attractive substrates for the design of biofunctional interfaces as some polymer brushes display particularly strong protein resistance,^45,191,192,200^ even in complex biological fluids, therefore enabling to promote selective binding and capture of biomarkers or cell adhesion for cell based assay design or tissue engineering applications.^13,17,148,149,201,202^ The control of these properties can be engineered through the design and selection of monomer chemistry, the architecture of the brush and substrate, the type of polymerisation technique selected and chemical approach used for biofunctionalisation.^203^ In this sense, biofunctionalization constitutes an important tool to confer bioactive properties, in which a variety of biomolecules can be chemically conjugated to polymer brushes, such as proteins, peptides, enzymes, among others.^204^ This section is intended as a guide allowing the identification of suitable strategies for polymer brush biofunctionalisation (Table 3 and Fig. 10).

**Table 3:** Overview of conjugation strategies to tether biomacromolecules to polymer brushes

Brush type	Bioactive compound	Biofunctionalization strategy	Functionalisation density	Application	Ref.
POEGMA-OH	GGGRGDS	NPC (*p*-nitrophenyl chloroformate)	0.5–12 pmol cm^−2^	Cell adhesion control	205
PHEMA	GGGRDGS peptides				
PHEMA	GGGRGDS, GGGRDGS, GGPHSRN and GGHRPSN peptides	NPC	8–23 pmol cm^−2^	Cell adhesion control	116
PGAMA	GFOGER-containing peptides	NPC	24 pmol cm^−2^	Cell adhesion control	206
POEGMA	RGD peptides	EDC/NHS	2 nmol cm^−2^	Cell adhesion control	207
PAA	RGD peptides	EDC/NHS	1.2–1.5 μg cm^−2^	Cell adhesion control	113, 208 and 209
Polyelectrolyte poly(*N*-(3-aminopropyl)methacrylamide hydrochloride)	Tet-20 peptides	Michael additions (maleimides)	1.7–4.0 nmol cm^−2^	Antimicrobial and antifouling properties	210
PGMA	CGGGRGDS peptides	Thiol–ene coupling	460 ng cm^−2^	Surface patterning and protein adsorption	67
POEGMA	RGD peptides	Thiol–ene coupling	n.s.	Cell adhesion control and migration	68
POEGMA-*b*-PGMA	Pep19-2.5 peptide	Azide–alkyne cycloaddition	300 ng cm^−2^	Antimicrobial and antifouling properties	202
PCBAA	Biotin/streptavidin	EDC/NHS	200 to 400–800 ng cm^−2^	Biosensor development	194
PCBAA	Biotin/streptavidin antibody conjugation	EDC/NHS	50–100 ng cm^−2^	Biosensor development	180 and 211
POEGMA-*co*-PHEMA	IgG antibody	EDC/NHS	130–680 ng cm^−2^	Biosensor development	212
PCBMAA-*co*-PHPMAA-*co*-PSBMAA	IgG antibody	EDC/NHS	150–220 ng cm^−2^	Biosensor development	56
POEGMA	Biotin/streptavidin antibody conjugation	EDC/NHS, DSC, NPC and other strategies	10–150 ng cm^−2^	Biosensor development	180
PHEMA					
POEGMA	AGT (angiotensin) protein	NPC/O6-benzylguanine	200–600 ng cm^−2^	Biosensor development	213

The combined hydrophilicity, crowding and protein resistance of polymer brushes makes the coupling of large macromolecules such as peptides or proteins challenging as they may not easily infiltrate or reside in close proximity with the brush for prolonged periods of time, reducing the likelihood of reactive chemical functions finding each other. This also has implications for the selection of the architecture of resulting constructs as macromolecule coupling may be restricted to the upper surface of the brush. Indeed, protein tethering to PGMA brushes was found to be restricted to the very top surface of the brush, based on X-ray photoelectron spectroscopy with depth profiling.^115^

As a result, the brush architecture was found to affect the coupling efficacy and specificity of functionalised polymer brushes. A biosensing platform based on IgG antibodies immobilized on POEGMA-*co*-PHEMA brushes *via* EDC/NHS coupling displayed increased IgG binding as the brush thickness and optimisation of their grafting density strongly impacted on antibody tethering. This was proposed to result from the optimisation of the density of coupling sites, whilst limiting non-specific protein adsorption.^28^ Similarly, poly(carboxybetaine acrylamide) brushes displaying a dense core compartment and a sparser, more extended outer compartment enabled high loading capacities and coupling of antibodies, whilst preserving the exceptional protein resistance of associated brushes.^194^

The chemistry selected for coupling of biomacromolecule to polymer brushes is an important design element to enable high functionality, without compromising on the retention of the brush physico-chemical properties. A broad range of chemical approaches have been proposed for the bioconjugation of polymer brushes, from coupling to carboxylic acids, hydroxyls and aldehydes to the application of thiol–ene chemistry, alkyne–azide cycloaddition and the use of motifs promoting supramolecular interactions. As these conjugation strategies overlap with those described for the functionalisation of brushes with small molecules, the focus of this section will be placed on particular features that impact the design of biofunctionalised polymer brushes.

### Peptides coupling to brushes

The presence of carboxylic acids and hydroxyl groups in the side chain of a broad range of polymer brushes, or at the end of their chains, has been routinely exploited for peptides conjugation. These strategies typically resulted in high peptide coupling and brush biofunctionality, although reactivities have not systematically been quantified. One of the advantages of peptide functionalisation, compared to protein or protein fragment coupling, is that peptide solutions can be prepared in anhydrous and aprotic solvents such as dimethylformamide, to avoid hydrolysis of activated esters and carbonates. Hence, hydroxyl functional polymer brushes such as POEGMA-OH or PHEMA have been activated with disuccinimidyl carbonate or nitrophenyl chloroformate, prior to direct reaction with peptides, such as cell adhesive peptides presenting RGD or GFOGER motifs.^205,206,214^ Corresponding peptides were typically directly coupled to brushes *via* their N-terminus, without further protection of arginine or serine residues. Surface densities of the peptides immobilised *via* such approach were investigated using fluorescently tagged peptides, with a range of 0.5–12 pmol cm^−2^ (approximately 0.5–12 ng cm^−2^) achieved.^205^ Considering the thickness of the brushes used for such characterisation and assuming a likely grafting density of 0.5 chains nm^−2^ and molecular weight of 50 kDa, these peptide densities correspond to relatively low coupling densities per chain (estimated in the range of 0.006–0.15 peptide per polymer chain). As these coatings were found to be bioactive and effective in promoting cell adhesion, this suggests that peptide functionalisation was confined to the apical compartment of the brush. This is also in agreement with the relatively strong peptide-specific signals detected for these functionalised brushes *via* XPS, a technique sensitive to the first 5–10 nm of the surface chemistry. This may be explained by the large size of the peptides of interest and their relatively slow diffusion into dense brushes (compared to small molecules with molar masses below 100 g mol^−1^). Comparable results were obtained (with slightly higher peptide densities of 8–23 pmol cm^−2^) in the case of PHEMA brushes.^116^

These results are in good agreement with GFOGER coupling densities achieved using a comparable brush and activation strategy, quantified vis SPR, in the range of 120 ng cm^−2^ (considering the molar mass of this peptide, this would correspond to 24 pmol cm^−2^).^206^ In contrast, the coupling of the GFOGER peptide to poly(2-gluconamidoethyl methacrylate) brushes resulted in 1 order of magnitude lower peptide densities (based on SPR data),^214^ perhaps as a result of less effective brush activation or peptide reactivity.

Similarly, EDC/NHS-mediated coupling was applied to the functionalisation of carboxylated POEGMA brushes with short RGD peptides, presumably *via* their N-terminus.^207^ Surprisingly, the density of tethered peptides reported *via* this approach was significantly higher than that reported for nitrophenyl chloroformate coupling to hydroxyl-POEGMA. Indeed, densities of 2 nmol cm^−2^, despite the functionalisation taking place in aqueous buffers with relatively fast hydrolysing activated esters. This coupling approach was also applied to the functionalisation of PMAA and PAA brushes and block copolymer brushes, although the resulting peptide density was not quantified.^117,195,215^ Poly(diethylene glycol methacrylate) brushes generated *via* RAFT polymerisation and presenting terminal carboxylic acid groups were also functionalised with short RGD peptides,^216^ promoting cell adhesion to levels comparable to those observed with block copolymers displaying greater densities of carboxylic function or to tissue culture polystyrene.^195,215^ Hence, although the efficiency of such end-chain functionalisation was not quantified, the peptide densities achieved must have been sufficiently high and potentially comparable to those obtained with brushes presenting multiple reactive functions.

Another approach proposed was to carry out nucleophilic substitutions onto iodoacetates, using thiols from cysteine residues.^217^*N*,*N*-Dimethyl acrylamide copolymer brushes with *N*-(3-aminopropyl)methacrylamide hydrochloride were functionalised with iodoacetic acid, using the corresponding *N*-hydroxysuccinimide ester, prior to coupling with C-terminal cysteine peptides *via* nucleophilic substitution. Although the peptide densities achieved were not quantified, XPS and FTIR confirmed the functionalisation. Catheters samples coated using this strategy with the antimicrobial polycationic peptide RRWRIVVIRVRRC displayed excellent antibacterial properties.

As peptides may display amino acids that are reactive towards activated ester and carbonate moieties (*i.e.* lysines, but also arginine, tyrosine and potentially serine residues, although to much lower levels), chemoselective reactions are attractive to specifically tether peptides *via* defined amino acids. In addition, the hydrolysis of intermediates may also be a limiting factor to the generation of functional brushes displaying high peptide densities. Therefore alternative strategies allowing the chemoselective coupling of peptide to brushes with high efficiencies are essential to explore.

The addition of thiols to maleimides through precisely positioned cysteines has received particular attention. To this end, maleimides introduced through thiocyanates reacting with hydroxyl-bearing brushes (*e.g.* POEGMA or PHEMA) were proposed. This enabled the coupling of the 23 amino acid antibacterial peptide Magainin I, for example.^218–220^ The lysine content and conformation of Magainin I are essential to retain in order to induce bacterial membrane porosity and death. Therefore the chemoselective tethering of this peptide is important to achieve. To further enhance the functional density of peptides at the surface of brushes, the polyelectrolyte poly(*N*-(3-aminopropyl)methacrylamide hydrochloride) was generated and functionalised with 3-maleimidopropionic acid *N*-hydroxysuccinimide, prior to reaction with various cysteine modified antibacterial peptides.^210^ This resulted in relatively high peptide densities (in the range of 10–24 peptide nm^−2^; corresponding to 1.7–4.0 nmol cm^−2^), implying a deep and extensive functionalisation. The peptide coupling densities measured were found to be significantly impacted by the grafting density of the brush (the brush grafting densities achieved, in the range of 0.03–0.15 chains nm^−2^, remained relatively low, compared to PHEMA/POEGMA brushes reported by others, closer to 0.5 chains nm^−2^).

Although the p*K*_a_ of lysines is relatively high (near 10.5), some cross-reactivity is possible, even at neutral pH, and depending on the impact of their environment (neighbouring amino acids). In addition, other nucleophilic residues such as arginines, tyrosines or even carboxylated amino acids may couple to activated alkenes such as maleimides *via* Michael addition. Therefore, radical thiol–ene and thiol–yne reactions have attracted some attention for functionalisation of hydrogels and interfaces.^104,221^ In aqueous buffers, at relatively low peptide concentrations typical of bioconjugation strategies, thiol–ene coupling was found to be significantly impacted by the pH of the environment, proceeding readily at neutral and low pH, in contrast to Michael addition reactions (which proceed in more alkaline pH).^145^ The presence of other amino acids was found to have relatively minor impact on the coupling efficiency, although the position of the cysteine underpinning the coupling did. This was proposed to result from a change in the p*K*_a_ of the cysteine, depending on its position on the peptide to be coupled: N-terminal cysteines display a lower p*K*_a_, resulting in poorer radical thiol–ene coupling at neutral pH, as the proportion of thiolate will be greater. Therefore, penultimate cysteines were proposed to be more effective for radical thiol–ene, whereas peptides displaying N-terminal cysteines are better suited to Michael additions.^145^

**Fig. 11:**
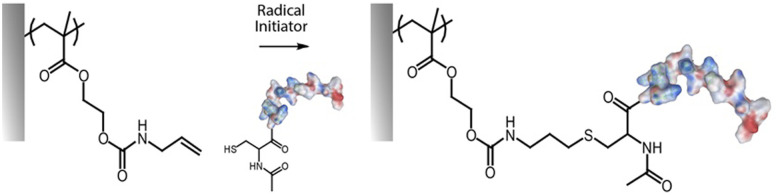
Example of thiol–ene radical coupling of a peptide (through a cysteine residue) to allylamine-functionalised PHEMA brushes.

The efficiency of thiol–ene radical coupling with short peptides (CGGGRGDS) to allylamine-functionalised PGMA brushes was found to be reduced compared to that observed for smaller thiols, such as acetyl cysteine.^67^ To some extent, this may be attributed to the terminal position of the cysteine used in this peptide, but is also the result of the crowding of the brush, as peptides seem to be primarily localised at the surface of the brush. Indeed, functionalisation levels determined by XPS (sensitive to the first 5–10 nm of the brush chemistry) were 14 ± 3%, whereas those determined from ellipsometry (quantifying changes occurring over the entire brush thickness) were only 6 ± 1%. These functionalisation levels correspond to peptide densities of 460 ng cm^−2^ (assuming a density of 1.4 g cm^−3^), significantly higher than those achieved by carbonate or activated ester coupling, but slightly under those reported for maleimide-based coupling. Potentially this may have resulted from the higher brush grafting density achieved for the PGMA brushes studied. Similarly, tethering levels achieved onto allylamine-functionalised PHEMA brushes (Fig. 11) and vinyl-terminated poly(oligo(2-alkyl-2-oxazoline)methacrylate) brushes were comparable, with peptide densities in the range of 280–620 ng cm^−2^.^67–69^ Measured coupling levels were lower, with norbornene-functionalised PHEMA brushes, but this was likely reflecting the reduced functionalisation of the brush with norbornene moieties, compared to that achieved with allylamine.^69^

Azide–alkyne cycloaddition has recently been applied for the chemoselective functionalisation of polymer brushes.^202^ POEGMA-*b*-PGMA block copolymers were conveniently functionalised with azides post-polymerisation, *via* simple incubation in sodium azide solutions, prior to coupling of a strained alkyne dibenzocyclooctyne-conjugated peptide. Such strained alkynes are able to undergo cycloaddition with azide spontaneously, without catalysis, at room temperature, in aqueous conditions and at relatively low concentrations.^222^ The resulting peptide coverage was relatively high (300 ng cm^−2^) and did not significantly impact the protein resistance of the POEGMA brush. Although cyclooctyne is typically relatively expensive and involves multi-step synthesis,^83^ other strained alkynes such as dibenzocyclooctyne (DBCO) and bicyclononyne (BCN) have seen increased popularity for bioconjugation.^137^ Interestingly, the immobilization of streptavidin was remarkably higher when using BCN compared to DBCO.^137^

Finally, another strategy proposed for the reversible functionalisation of polymer brushes with cell adhesive peptides consists in capturing RGD-functionalised poly(3-gluconamidopropyl methacrylamide), and RGD peptides presenting 3,4-dihydroxyphenylalanine (DOPA) tetramers, with phenylboronic acid-functionalised polymer brushes.^223,224^ Hence PHEMA and poly(2-hydroxyethyl acrylamide) were functionalised with phenylboronic acid moieties using dicyclohexyl carbodiimide as coupling agent, to allow the capture of RGD-functional macromolecules based on boronic acid–diol interactions that can be displaced through the introduction of competitors such as glucose. This enabled promoting cell adhesion and selective cell detachment. Similarly, vancomycin-conjugated polymer brushes allowed the reversible capture of cell adhesive peptide-functionalised macromolecules presenting multiple alanine dimers.^225^ These strategies are attractive to confer dynamic bioactive properties to corresponding interfaces, to modulate cell adhesion, a strategy potentially applicable to a broader range of ligands to engage cell membrane receptors.

### Protein coupling to brushes

A similar, if not greater, range of chemical coupling strategies has been applied to the functionalisation of polymer brushes with proteins, compared to peptides. However, protein tethering requires additional considerations to be taken into account. First, most proteins require coupling in aqueous conditions, which are often not essential for peptide solubilisation, yet may impact the (hydrolytic) stability of reactive groups. Second, the higher molecular weight of proteins is associated with reduced infiltration into brushes and associated residence time for coupling, compared to peptides or small molecules. This may also result in predominantly apical tethering to brushes,^115^ with little evidence of protein coupling within brushes, although the brushes for which protein localisation was explored was relatively hydrophobic and may not swell sufficiently for extensive infiltration to occur. Third, requirements for chemoselectivity and oriented coupling are even greater for proteins than for peptides, in order to preserve the bioactivity of associated macromolecules.

The coupling of proteins to brushes *via* activated esters and carbonates has been widely studied. A direct comparison of a broad range of different strategies for the functionalisation of POEGMA brushes (hydroxyl side-chain terminated) revealed that comparable levels of tethering of streptavidin to brushes activated with cyanuric chloride, carbonyldiimidazole (CDI), triflic anhydride and EDC/NHS (after functionalisation with succinic anhydride) were observed, whereas coupling with disuccinimidyl carbonate was found to be higher.^180^ This was despite the increased protein non-specific fouling (including from streptavidin) observed for carboxylated brushes. As the density of functional groups and the grafting density of polymer brushes compared was identical in this study, these parameters cannot account for such difference. Beyond differences in reactivity between succinimidyl carbonates and esters, it could be proposed that the significantly longer lifetime of carbonate intermediates may provide greater opportunities for coupling. Indeed, whereas the half-life of EDC/NHS activated esters is only 10 min at room temperature and neutral pH,^45,46^ carbonates can persist for hours and even days in similar conditions and could be seen post-streptavidin functionalisation in XPS spectra.^180^ However, carbodiimide chemistry has been used to covalently couple glucose oxidase to PAA brushes, enabling higher levels of immobilisation compared to physical adsorption.^226^ This led to immobilisation levels near 300 ng cm^−2^.

Poly(zwitterionic) brushes based on carboxylic acid residues can provide an attractive approach to remediate the issue associated with the conflict between protein resistance (preventing protein infiltration and long dwell times in the vicinity of reactive groups) and efficient coupling. Indeed, upon simple activation with EDC/NHS, the zwitterionic residues of brushes such as poly(carboxybetaine acrylamide) convert to positively charged moieties that can promote the strong attraction of many proteins, in near-neutral conditions.^45^ However, upon gradual hydrolysis of unreacted moieties, during subsequent incubation in aqueous solutions, these residues revert to zwitterions. This leads to the excellent preservation of the ultra-low fouling of such brushes, yet high protein functionalisation levels for enhanced biosensing applications, for example. Protein functionalisation levels were found to be in the range of 200–250 ng cm^−2^, in the case of antibodies against *Salmonella sp.* or Thyroid Stimulating Hormone, and for neutravidin, whereas streptavidin led to weaker adsorption (120 ng cm^−2^), perhaps due to the higher level of glycosylation of this protein.^45,194,211^ Recently, a biosensing platform for detection of SARS-CoV-2 was also proposed based on antibodies conjugated to poly(*N*-(2-hydroxypropyl) methacrylamide)-*r*-poly(carboxybetaine methacrylamide)-*r*-poly(sulfobetaine methacrylamide) brushes, using the same EDC/NHS functionalisation strategy.^56^ This approach has also found application for the coupling of proteins involved in the breakdown of blood clots^57^ and antibodies recognising the coagulation Factor XII (FXIIa)^227^ for hemocompatible coatings design. Similarly, IgG antibodies were coupled to terpolymer brushes *via* EDC/NHS and displayed surface densities in the range of 150–220 ng cm^−2^.^56^

The architecture of brushes had a strong impact on protein coupling. This was mainly investigated in the context of antibody coupling, for the biomonitoring of various markers. Hence, re-initiation of poly(carboxybetaine acrylamide) brushes, with reduced grafting densities, led to the retention of the excellent protein resistance of the first underlying block, whilst reducing crowding in the upper block and enabling the infiltration of proteins to achieve increased coupling densities.^194^ Depending on the level of grafting density and length of the re-initiated polymers, the density of protein adsorbed increased from approximately 200 ng cm^−2^ to 400–800 ng cm^−2^. In contrast, re-initiation of methoxy-POEGMA brushes (unreactive) with hydroxy-POEGMA (for disuccinimidyl carbonate coupling) led to a reduction in the tethering of proteins, depending on the size of the reinitiated second block.^211^ When poly(carboxybetaine acrylamide) was re-initiated, comparable levels of protein tethering were observed compared to the direct coupling to a single block of this brush. However, when copolymer brushes of POEGMA (unreactive) and PHEMA were grafted at low density, with an oligoethylene glycol spacer to retain protein resistance, relatively high protein densities were achieved, in the range of 130–680 ng cm^−2^.^212^ Hence, overall, a more open and sparse architecture seems beneficial to promote protein loading, providing the associated loss in protein resistance can be offset.

**Fig. 12:**
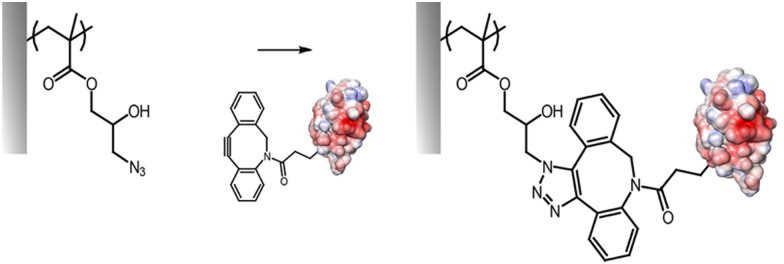
Example of strain-induced azide–alkyne cycloaddition for the coupling of a cyclooctyne-conjugated protein to azide-functionalised brushes.

An alternative strategy to covalent coupling, to enable protein coupling to anti-fouling brushes, consists in using host–guest interactions or ligand complexation. Biotin–streptavidin binding, as for other biointerfaces and biomaterials, has been often exploited for the tethering of biotinylated proteins and antibodies to polymer brushes. Two main strategies can be developed. In the first, brushes are directly biotinylated, as side-chain or end-chain residues, prior to streptavidin binding.^180^ In the second, brushes are first streptavidinated, prior to binding of biotinylated biomacromolecules.^180,211^ The high affinity constant associated with biotin–streptavidin binding, together with the excellent stability of both molecular components is presumably essential to enable the reasonable protein densities achieved (in the range of 50–100 ng cm^−2^). However, these levels remain under those measured for direct protein adsorption, suggesting that ligand availability and increased crowding upon successive protein adsorption events is resulting in reduced loading capacities. More recently, the use of azide–alkyne cycloaddition was also introduced to promote protein coupling to polymer brushes (Fig. 12). Hierarchically structured copolymer brushes, for example, can combine antifouling polymer bottom block and an azide-functional upper blocks.^189^ This approach led to comparable levels of protein tethering to resulting brushes, compared to other methods of biotinylation.

Although the functionalisation strategy and architecture of brushes was found to have an important impact on the density of proteins primarily coupled to these interfaces, subsequent binding of proteins was found to be less affected by such design. Hence the level of proteins captured by antibody-functionalised polymer brushes was typically reduced, compared to the primary antibody immobilisation, and different strategies that had led to substantial changes in antibody tethering led to relatively similar analyte detection.^180,211^ This suggests that crowding at the surface of the brush is an important factor impacting on recognition and associated maximum binding capacity. Hence, different length of side chains or upper blocks in block copolymer structures did not affect significantly secondary binding.^180,211^ It is likely that the identification of smaller recognition motifs may improve on the density of markers that can be bound, by reducing crowding at the brush surface, and enhance the sensitivity of corresponding bioassays. Other potential strategies may include “sandwich” assays, with high mass or signal amplification.

Finally, other strategies have been proposed for the selective oriented coupling of proteins to brushes. Indeed, even with streptavidinated brushes, biotinylated proteins rarely offer opportunities for controlling the orientation and the precise presentation of binding motifs. This is because the biotinylation of these proteins or antibodies is itself not specific to one amino acid. It is possible to introduce specific tags in recombinant proteins (*e.g.* Avi-tag, for conversion to biotin residues), but these tools have not been systematically implemented for the functionalisation of brushes. One of the most widely used tags in the context of brushes is the histidine tag, able to bind nitrilotriacetate-Ni^2+^ complexes decorating the structure of brushes.^49,52,124,228^ This was found to result in relatively high protein loading levels (200–600 ng cm^−2^), although the stability of the complexes formed is a limiting factor. The formation of selective covalent bonds between brushes and target proteins was achieved using *O*^6^-alkylguanine–DNA-alkyltransferase (AGT) fusion proteins that specifically coupled to *O*^6^-benzylguanine (BG) residues coupled to POEGMA brushes.^213^ Combining such tools with recombinant protein design and expression will confer further specificity and precision to the biofunctionality of brush-based interfaces.

## Conclusions

The field of polymer brush design has seen significant development of a broad range of synthetic tools, not only to control the architecture of polymer brushes and their patterning, but also for the conjugation of a wide range of molecules, peptides, proteins and antibodies. In addition to significant progress in the translation and scale up polymer brush growth processes and the improvement of the stability of these coatings, this flexibility of design will have an impact on a broad range of applications. The versatility, complementarity and orthogonality of some of the functionalisation strategies developed will contribute to expand the complexity of tailored brushes and the optimisation of their properties, for example for the design of multi-functional interfaces and responsive dynamic surfaces and associated materials. However, challenges remain in particular for the incorporation of relatively sensitive molecules at high densities, or for the chemoselective tethering of peptides and proteins at high densities. Similarly, tools enabling the precise structuring of the chemistry and functionality of polymer brushes will enable the engineering of nanostructured soft materials that can rival in complexity and specificity with protein assemblies regulating a range of biological processes and for therapeutics delivery. Nevertheless, important design guidelines have emerged and will contribute to expand the range of applications of polymer brushes and their translation.

## Conflicts of interest

The authors declare no conflict of interest.

## Supplementary Material
